# The role of H_2_S and its derivatives in plant responses to low O_2_

**DOI:** 10.1093/jxb/erag402

**Published:** 2026-08-21

**Authors:** Yuri Telara, Salma Akter, Emily Flashman, Beatrice Giuntoli

**Affiliations:** Biology Department, University of Pisa, via L. Ghini 13, 56126 Pisa, Italy; Department of Biology, University of Oxford, South Parks Road, Oxford OX1 3EL, UK; Department of Biology, University of Oxford, South Parks Road, Oxford OX1 3EL, UK; Biology Department, University of Pisa, via L. Ghini 13, 56126 Pisa, Italy; Fukui Prefectural University, Japan

**Keywords:** Flooding stresses, gasotransmitters, hydrogen sulfide, hypoxia, oxygen sensing, persulfidation, plant cysteine oxidase

## Abstract

Hydrogen sulfide (H_2_S) is a gasotransmitter molecule that regulates essential plant biological processes. In the context of climate change and increasing flooding events, its role in plant responses to oxygen (O_2_) deficiency has gained significant attention. Accumulating evidence indicates that H_2_S exerts beneficial effects under hypoxia by modulating antioxidant defenses, mitochondrial function, and hormonal signaling, mainly through its ability to refine protein activity via the post-translational modification (PTM) of persulfidation. Here, we discuss how this PTM is linked to the gasotransmitter molecule nitric oxide (NO) and reactive oxygen species (ROS), and how they can act synergistically or antagonistically, influencing the cell redox state and survival. Additionally, we examine how H_2_S turnover is intrinsically linked to O_2_ availability, acting as a conserved mediator of low O_2_ responses in different domains of life. By integrating biochemical insights with molecular signaling, we underscore the potential of H_2_S in enhancing crop resilience to low O_2_-related stresses, such as submergence and waterlogging.

## Teaser

Through a comprehensive and critical evaluation of the available literature, we compare and discuss contrasting data to better define the molecular mechanisms through which H_2_S may influence low-oxygen signaling pathways.

## Introduction

Climate change is increasing the frequency of environmental disasters, which have a devastating effect on global agriculture. A recent report from the United Nations Food and Agriculture Organization states that from 1991 to 2023, disasters caused US$3.6 trillion damage, with flooding being the most economically destructive hazard (US$1.5 trillion losses over the same period) (FAO, 2025). Strategies to improve crop resilience to flooding are necessary to mitigate crop losses, which can be facilitated by an understanding of plant molecular responses to submergence.

A significant stressor upon plant submergence is the reduction in O_2_ availability, which limits aerobic respiration and efficient ATP production. The molecular response to reduced O_2_ availability, which directs a switch to anaerobic metabolism, has been well characterized and is described in detail in many recent reviews (Voesenek and Bailey-Serres, 2015; Loreti and Perata, 2023; Renziehausen *et al*., 2025). However, flooding is a compound stress that includes the accumulation of other gaseous signals, as well as signals initiated by other factors such as darkness and energy availability (Renziehausen *et al*., 2024). Major gaseous compounds involved in cell signaling during flooding are nitric oxide (NO) and the hormone ethylene (Sasidharan *et al*., 2018; Hartman *et al*., 2019). Lately, a role is emerging for a third molecule, hydrogen sulfide (H_2_S).

H_2_S (also termed sulfane) is a soluble, volatile and cell-permeable gas which is toxic at high concentrations. It is only in the last 30 years that it has been revealed to have a role in cell physiology (Wang *et al*., 2022). Although H_2_S itself does not accumulate to high concentrations, H_2_S-derived signals can affect a range of physiological processes which, in plants, have been reported to act as important signals in development, growth, and stress responses, described in detail in several reviews (Paul and Snyder, 2012; Aroca *et al*., 2015; Gotor *et al*., 2019; Moseler *et al*., 2024). Some cornerstone proteomic studies have demonstrated that protein post-translational modification (PTM) is the main mechanism for H_2_S-mediated signaling in plants (Aroca *et al*., 2017, 2020; Romero *et al*., 2026). Persulfidation can affect the localization, conformation, dimerization, or activity of proteins, according to its particular targets, thereby exerting a range of profound effects on cell regulation (Zhou *et al*., 2025).

The close relationship between O_2_ availability and H_2_S oxidation suggests that H_2_S-derived signals may be indicators of hypoxia and oxidative stress. However, the dynamic reactivity of H_2_S-derived species, including with prevalent intracellular thiols such as Cys residues and glutathione (GSH), makes these modifications challenging to study. The modes of action of H_2_S-based signaling are therefore not yet fully understood. In this review, we summarize current understanding of the role of H_2_S in plant physiology, with a focus on its involvement in low O_2_ signaling. We discuss the range of H_2_S-dependent PTMs and their dynamics as well as how these are affected by changes in O_2_ availability and redox status. Finally, we propose that H_2_S-derived signals may play a more prevalent role in dynamic plant stress responses than previously thought. However, careful analysis and accurate characterization of H_2_S-derived persulfidation reactions are essential to reconcile the complex chemical and biological features of this fascinating molecule.

## Hydrogen sulfide as a gasotransmitter

### From toxic molecule to gaseous signal

Gasotransmitters are small, endogenously produced gaseous molecules able to serve as signaling messengers (the neologism first appeared in an article referring precisely to H_2_S, as a contraction of ‘gaseous transmitters’; Wang, 2002). The concept has emerged from a series of discoveries that revolutionized cell biology in the late 20th century. For decades, gases such as NO, carbon monoxide (CO), and H_2_S have been considered environmental pollutants or metabolic byproducts rather than signaling molecules. The turning point came in the 1980s, when NO enzymatic synthesis by nitric oxide synthase (NOS) was demonstrated and its functions as a key regulator in human physiology were identified, as a mediator of the relaxation of vascular smooth muscle caused by endothelium-derived relaxing factor (EDRF) (Ignarro *et al*., 1987). These findings earned the authors the 1998 Nobel Prize in Physiology or Medicine and introduced the notion that small gaseous molecules can act as signaling messengers.

Following this conceptual breakthrough, researchers began exploring whether other gas molecules could serve a role in signaling. H_2_S, long recognized for its characteristic odor and toxicity, became a candidate. It was reported in 1996 that brain tissues produce measurable H_2_S levels and that this can modulate *N*-methyl-D-aspartate (NMDA) receptor activity, marking the first evidence of H_2_S-derived signals as a neuromodulator (Abe and Kimura, 1996). Subsequent work has identified cystathionine β-synthase (CBS), cystathionine γ-lyase (CSE), and 3-mercaptopyruvate sulfurtransferase (3-MST) as enzymes responsible for H_2_S biosynthesis in mammals (Hosoki *et al*., 1997). More recently, the H_2_S-producing activity of the methanethiol oxidase (MTO) SELENBP1 has been discovered in humans (Pol *et al*., 2018). By the early 2000s, H_2_S had thus been recognized as the third gasotransmitter in humans, following NO and CO (Wang, 2012). The chemical and physical properties of these molecules allow them to diffuse through membranes and act independently of vesicular trafficking or receptor binding (Mustafa *et al.*, 2009b). They can accumulate locally, integrating environmental and metabolic stimuli into cellular signaling (Mustafa *et al.*, 2009b). Moreover, another distinctive feature of gasotransmitters is their high instability and chemical reactivity (Wang, 2014).

In plants too, NO, CO, and H_2_S are established gasotransmitters, involved in stress responses and development (Kolupaev *et al*., 2022). The identification of methane and molecular hydrogen (H_2_) as additional gasotransmitters, both found to be connected to stress responses, is presently hindered by the lack of an identified mechanism of biosynthesis and action in plants (Yao *et al*., 2019; L. Li *et al*., 2020). Ethylene is another gas ubiquitously generated by plants that covers paramount functions in signaling. Despite this, ethylene, being a phytohormone, rather eludes the definition that describes gasotransmitters as reactive molecules that function independently of receptors (Wang, 2014). The discovery of H_2_S as a plant gasotransmitter, only preceded by the identification of NO, dates back to the observation of H_2_S emissions from leaves of several plant species (Wilson *et al*., 1978). As in animal systems, H_2_S in plants meets the defining criteria of being a small membrane-permeable gas, generated by endogenous and regulated enzymatic pathways, having high reactivity, and interacting with signaling pathways (directly or with the mediation of hormones) (Wang, 2002). Overwhelming evidence has built up over time that shows H_2_S participation in numerous signaling pathways (Aroca *et al*., 2020; Xuan *et al*., 2020; Wang *et al*., 2022) during stress and growth responses that are mediated, among other signals, by hormones (Fang *et al*., 2014; Aroca *et al*., 2021; Yuan *et al*., 2025), Ca^2+^ (Fang *et al*., 2017), NO (Corpas *et al*., 2019; Mukherjee and Corpas, 2020), and reactive oxygen species (ROS) (Shen *et al*., 2020; Xiao *et al*., 2020).

### Biosynthesis and signaling role of hydrogen sulfide in plants

H_2_S in plants is mainly produced by cysteine desulfhydrase enzymes (CDes) (Rennenberg *et al*., 1987), including L-cysteine desulfhydrase (LCD) and the bifunctional cystathionine γ-lyase/cysteine synthase [DES1, a member of the *O*-acetylserine thiol lyase (OASTL) family]. CDes decompose L-Cys into H_2_S, pyruvate, and NH_4_^+^ using pyridoxal 5'-phosphate (PLP) as cofactor. Among them, DES1 is a cytosolic enzyme (Álvarez *et al*., 2010), while the LCD family encompasses both cytosolic and nuclear isoforms (González-Gordo *et al*., 2020; Hu *et al*., 2020; Cao *et al*., 2022) (Fig. 1). Chloroplasts can also generate H_2_S through sulfite reductase (SiR), during the reductive steps of sulfate assimilation (Fig. 1). At the alkaline pH typical of the stromatic environment under illumination, H_2_S (p*K*_a_=6.98) (Vignane and Filipovic, 2023) predominantly exists as soluble hydrosulfide (HS^−^) and is unable to cross the chloroplast envelope in the absence of dedicated transporters (Gotor *et al*., 2019). Hence, H_2_S release from this organelle is believed to be negligible, but the molecule can still have a broad impact on the regulation of plastidial pathways (Aroca *et al*., 2015; Corpas *et al*., 2019). A third source of H_2_S is represented by the mitochondrial β-cyanoalanine synthases (β-CASs), such as CYS-C1 in Arabidopsis, the prevalent isoform in this family (Watanabe *et al*., 2008). β-CASs, PLP-dependent enzymes that convert Cys and cyanide to H_2_S and β-cyanoalanine, are required to maintain endogenous cyanide at low levels compatible with physiological processes (Machingura *et al*., 2016) (Fig. 1). However, the option that insufficient H_2_S production could contribute to the alterations observed in β-CAS mutants during root hair formation or salt stress responses has not been addressed so far (García-Mata and Lamattina, 2010; Xu *et al*., 2023).

**Fig. 1.**
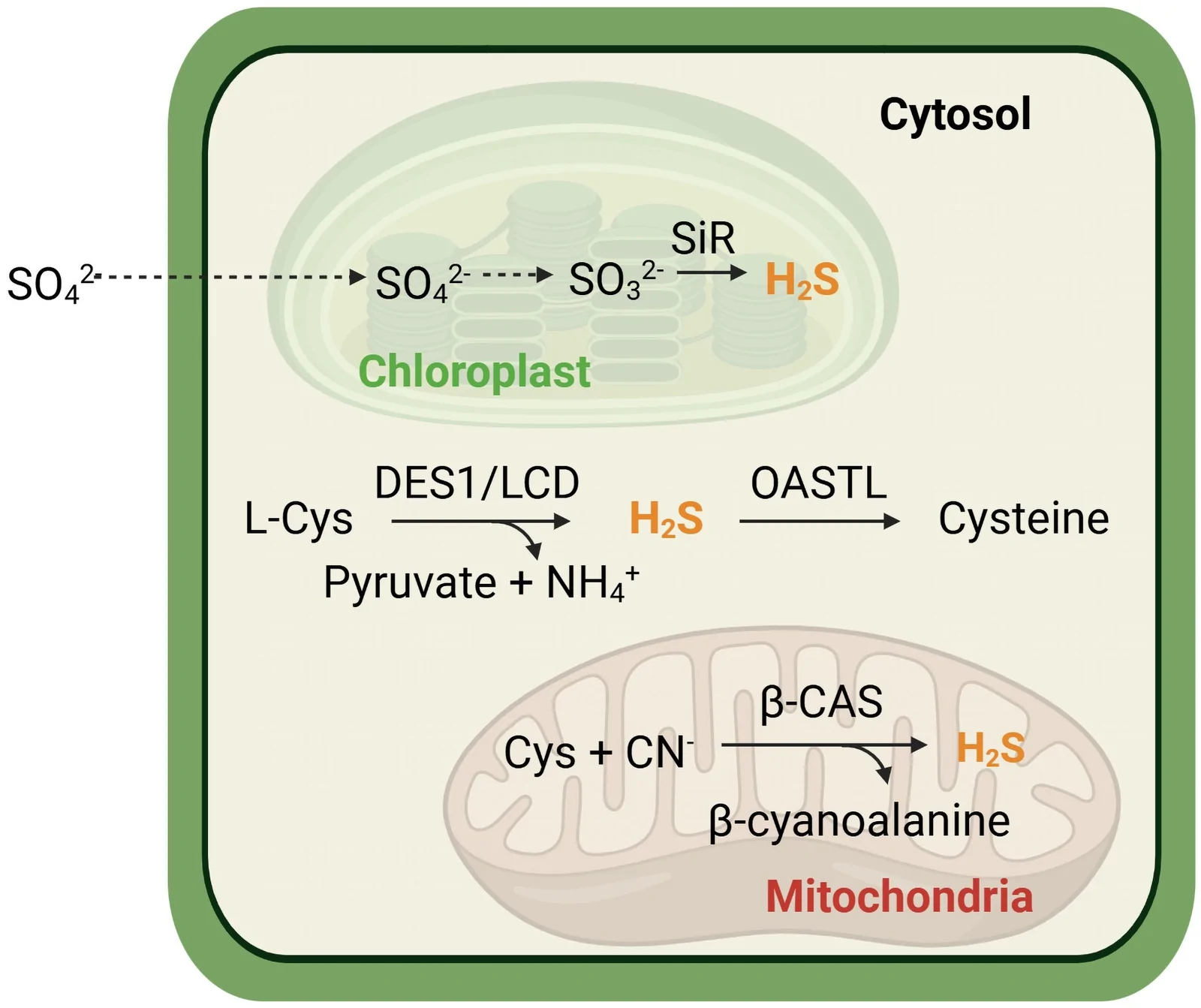
H_2_S biosynthesis in plant cells. H_2_S can be released in different cell compartments, with cytosolic desulfhydration being the prevalent pathway. SO_4_^2−^, sulfate; SO_4_^3−^, sulfite; NH_4_^+^, ammonia; CN^−^, cyanide; SiR, sulfite reductase (*AT5G04590*); DES1, L-cysteine desulfhydrase 1 (*AT5G28030*); LCD, L-cysteine desulfhydrase (*AT5G28020*); OASTL, *O*-acetylserine(thiol)lyase isoform A1 (*AT4G14880*); β-CAS, β-cyanoalanine synthase C1 (*AT3G61440*). Dotted lines represent multistep processes. Created in BioRender. Flashman, E. (2026) https://BioRender.com/oy343jm.

Among the H_2_S-producing routes, the DES1 pathway (the most recently identified pathway) is recognized as the prevalent source in plants, accounting for ∼30% H_2_S production in Arabidopsis tissues (Álvarez *et al*., 2010; Aroca *et al*., 2017; Gotor *et al*., 2019). Knockout *des1* mutants show early leaf senescence, enhanced defense responses against biotrophic and necrotrophic pathogens, and lower susceptibility to cadmium toxicity (Álvarez *et al*., 2010, 2012a). The isolation of *des1* has paved the way for numerous studies that have demonstrated the involvement of H_2_S in senescence, autophagy, stomatal closure, and immunity (Álvarez *et al*., 2010, 2012b; Scuffi *et al*., 2014; Laureano-Marín *et al*., 2016).

Part of the observed effects associated with the manipulation of H_2_S levels have been related to Cys homeostasis and antioxidant responses. Low Cys desulfuration in *des1* knockout plants leads to higher Cys content (Álvarez *et al*., 2010), while on the other hand Cys biosynthesis through OASTL action is known as a detoxification mechanism in the presence of excessive H_2_S levels (Birke *et al*., 2015). The interplay between H_2_S and Cys has consequences for redox homeostasis, through both GSH and the regulation of antioxidant enzyme activity. GSH is a Cys-containing tripeptide that acts as a primary antioxidant and key player in the redox balancing of cells (Noctor *et al*., 2011). The amelioration of plant survival promoted by both Cys and H_2_S supplementation in the presence of heavy metal stresses can be partially explained by an improvement in redox homeostasis associated with higher Cys production, in addition to the synthesis of Cys-derived metal chelators, such as phytochelatins and metallothioneins (Fang *et al*., 2016, 2017; Jia *et al*., 2016). Nonetheless, the role of H_2_S appears to go well beyond its participation in a metabolic feedback loop with Cys. H_2_S- and Cys-treated Arabidopsis seedlings showed limited transcriptional overlap and H_2_S-specific responses associated with transcriptional regulation, PTMs, and hormone signal transduction (Fang *et al*., 2023). This suggests that the mode of action of H_2_S entails Cys-independent mechanisms, with protein persulfidation considered as the predominant mechanism by which H_2_S-mediated signaling takes place (Vignane and Filipovic, 2023).

## Post-translational protein modifications associated with hydrogen sulfide

H_2_S, along with its oxidized derivatives, belongs to a broader group known as reactive sulfur species (RSS), a collective class of sulfur-containing compounds, analogous to ROS and reactive nitrogen species (RNS) (Cortese-Krott *et al*., 2017). Over the past two decades, RSS have been established as regulated mediators of redox signaling and oxidative stress, positioning sulfur as a central component of redox biology (Giles *et al*., 2017). The capacity of sulfur to span oxidation states from −2 (thiols, H_2_S) to +6 (sulfate) (Fig. 2A) confers functional versatility, permitting sulfur-containing molecules to mediate diverse redox processes (Mishanina *et al*., 2015; Smith and Pluth, 2023). In cells, sulfur in oxidation state −2 functions primarily as a reductant and nucleophile. At −1 and 0, it forms disulfides (-SS), polysulfides (-SS*_n_*-R), persulfides (-SSH), sulfenic acids (-SOH), and *S*-nitrosothiols (-SNO). These redox-dependent modifications, promoted in the presence of ROS, RNS, and/or RSS, play active roles in redox signaling. At higher oxidation states, sulfur occurs as sulfinic acids (-SO_2_H), sulfonic acids (-SO_3_H), and sulfate (SO_4_^2−^), which are generally regarded as terminal oxidation products, with the notable exception of target-specific sulfinic acid reduction mediated by sulfiredoxin (Biteau *et al*., 2003; Mishanina *et al*., 2015; Akter *et al*., 2018; Filipovic *et al*., 2018).

**Fig. 2.**
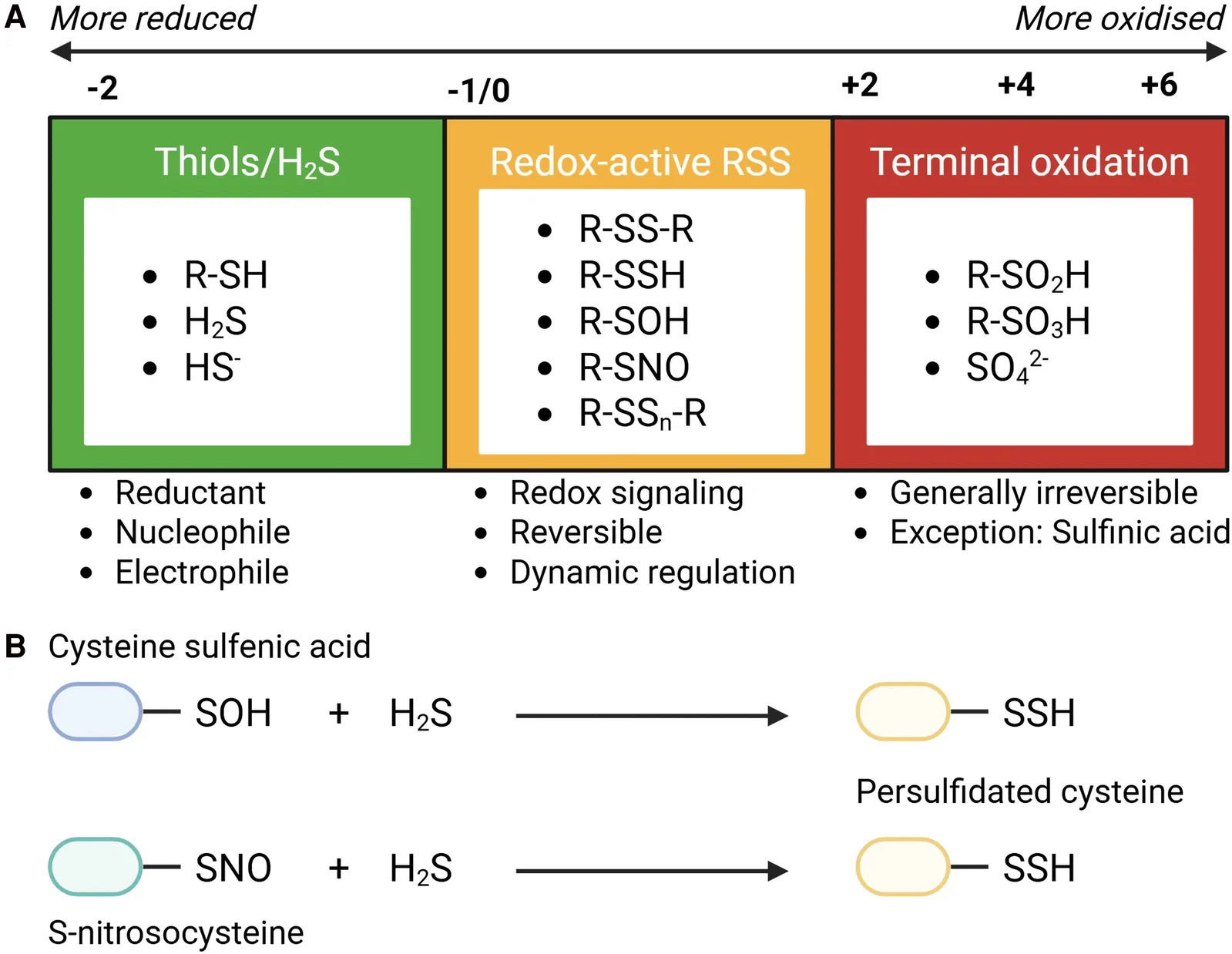
Oxidation states of biologically relevant sulfur species and their functional roles. (A) Sulfur spans oxidation states from −2 to +6, enabling participation in reductive chemistry, reversible redox signaling via reactive sulfur species (RSS), and terminal oxidative products. Among the terminal oxidation states of sulfur, sulfinic acids represent an exception to irreversibility, as Cys-sulfinic residues can be reduced through target-specific sulfiredoxins. (B) Persulfidated Cys is usually formed after H_2_S reaction with Cys sulfenic acid or *S*-nitrosocysteine residues in proteins. R-SH, thiol group; H_2_S, hydrogen sulfide; HS^−^, hydrosulfide anion; R-SS-R, disulfide bond; R-SSH, persulfide group; R-SOH, sulfenic acid; R-SNO, *S*-nitrosothiol; R-SS*_n_*-R, polysulfide chain; R-SO_2_-H, sulfinic acid; R-SO_3_-H, sulfonic acid; SO_4_^2−^, sulfate ion. Created in BioRender. Giuntoli, B. (2026) https://BioRender.com/ush26ff.

Within the cellular redox landscape, H_2_S is the most reduced biologically relevant sulfur species. It is maintained at low intracellular concentrations (∼10–30 nM in mammalian systems), consistent with its role as a tightly regulated and spatially confined signaling molecule (Furne *et al*., 2008; Vitvitsky *et al*., 2012; Kabil *et al*., 2014). H_2_S is a weak acid and, in aqueous solutions at physiological pH, is present mainly as hydrosulfide anion (HS^−^), while recent studies indicate that S^2−^ does not exist in measurable amounts in aqueous solutions (May *et al*., 2018). Under these conditions, HS^−^ can function either as a ligand or as a redox-active sulfur donor, depending on the reaction context.

O_2_ availability is a key determinant of H_2_S turnover. Under aerobic conditions, H_2_S is rapidly consumed through mitochondrial oxidation pathways and thiol-dependent reactions that promote its conversion into persulfides and other more oxidized forms (Olson, 2012; Filipovic *et al*., 2018). In contrast, under anaerobic or hypoxic conditions, oxidative H_2_S catabolism is suppressed, resulting in lower mitochondrial and O_2_-dependent turnover, greater persistence of H_2_S/HS^−^, and diminished formation of RSS. This is consistent with a model in which H_2_S and RSS metabolism is inversely correlated with O_2_ tension and has led to the proposal that H_2_S-derived reactions can act as an indirect O_2_-sensing mechanism, at least in animal systems (Olson, 2021). The cellular redox state further governs reaction selectivity, as oxidizing environments favour reaction between H_2_S/HS^−^ and more oxidized thiol species, thereby shaping RSS formation and downstream redox signaling processes (Cuevasanta *et al*., 2015; Wedmann *et al*., 2016).

H_2_S interacts with proteins through two mechanistically distinct chemical processes: (i) reversible coordination of HS^−^ with iron centers in heme-containing proteins; and (ii) covalent modification of Cys residues via persulfidation. At physiological pH, HS^−^ readily coordinates with Fe^2+^/Fe^3+^ centers in proteins, forming transient ferric sulfide complexes that are unstable under aerobic conditions, as shown for the human hemoglobin, myoglobin, neuroglobin, and cytochrome *c* proteins (Jensen and Fago, 2018; Vitvitsky *et al*., 2018). Here, these complexes undergo oxidation, converting sulfide (−2) to thiosulfate (with sulfur in mixed −1/+5 oxidation states) and to metal-bound polysulfides (sulfur in mixed −1/0 oxidation states). These interactions can thereby modulate local H_2_S availability while also generating RSS that can participate in redox signaling and antioxidant defense (Vitvitsky *et al*., 2015; Ruetz *et al*., 2017).

In parallel, persulfidation involves the reversible conversion of protein cysteine thiols (-SH, −2) into persulfides (-SSH, −1), a post-translational modification that enhances sulfur nucleophilicity, lowers the apparent thiol p*K*_a_, and can protect Cys residues from irreversible overoxidation to -SO_2_H or -SO_3_H. Through these properties, persulfidation functions as a dynamic redox switch in cellular signaling (Mustafa *et al*., 2009a; Wedmann *et al*., 2016; Filipovic *et al*., 2018). Primary representatives of persulfidation are per- and polysulfides (R-SS*_n_*H), where the R group denotes protein cysteine side chains and *n* is ≥1. Persulfidation can also occur on GSH and other low-molecular-weight thiols, contributing to the broader cellular persulfide pool (Ida *et al*., 2014). Importantly, H_2_S cannot react directly with other reduced thiols, as the direct reaction is both thermodynamically unfavorable and kinetically slow. From a mechanistic perspective, a need for oxidative priming is consistent with the low reactivity of H_2_S toward reduced thiols. Instead, persulfidation typically proceeds via oxidized Cys intermediates, such as sulfenic acids, disulfides, or *S*-nitrosothiols (Fig. 2B), or through reactions with other reactive sulfur species and polysulfides. Chemical and cellular studies support this model and show that persulfidation can act as a protective modification. Sulfenylation (the conversion of cysteine thiols into their first oxidized state, -SOH) is a reversible modification that regulates protein folding and function (Beedle *et al*., 2016; Huang *et al*., 2019). The unstable -SOH groups can be rapidly oxidized to Cys-sulfinic or -sulfonic acids, the latter at least being an irreversible process that is associated with protein oxidative damage. By resolving ROS-mediated sulfenylation, persulfidation prevents irreversible Cys overoxidation and helps preserve protein function under stress conditions (Filipovic *et al*., 2018; Zivanovic *et al*., 2019; Andrés *et al*., 2025). The rate constant for persulfide formation from H_2_S and sulfenic acids (-SOH) is 270 M^−1^ s^−1^, which is markedly higher than that for reactions with disulfides (0.6 M^−1^ s^−1^). This kinetic difference indicates that protein persulfidation probably proceeds primarily through the reaction of H_2_S with sulfenic acid groups. As indicated above, persulfidation modifications are subsequently reversible and tightly regulated by thioredoxin-, glutaredoxin-, and GSH-dependent systems (Dóka *et al*., 2016; Moseler *et al*., 2024). Notably, this pathway is kinetically favored *in vivo* because protein cysteine thiols are far more abundant than H_2_S, and these thiols readily react with ROS, particularly H_2_O_2_, at rates that are orders of magnitude faster than with sulfide. Consequently, ROS act as critical facilitators of H_2_S-mediated redox signaling by generating the oxidized thiol intermediates required for persulfidation (Filipovic *et al*., 2018; Benchoam *et al*., 2019; Zivanovic *et al*., 2019; Liu *et al*., 2024). H_2_S-derived signaling via protein modification is therefore complex and must be experimentally analyzed with care and attention to the redox conditions in both the biological context and methodology (Li and Lancaster, 2013).

## Protein persulfidation in plant physiology

### Persulfidation targets in plant signaling and stress responses

Proteomic analyses show that protein persulfidation is a widespread PTM, affecting hundreds of proteins in mammalian systems and thousands in plants. Identified targets span central metabolism, antioxidant defense, transcriptional regulation, and signaling pathways, underscoring the broad regulatory scope of persulfidation in plant physiology (García-Mata and Lamattina, 2010; Aroca *et al*., 2017; Li *et al*., 2024). A major group of targets includes metabolic enzymes that coordinate energy and redox balance. For example, cytosolic glyceraldehyde-3-phosphate dehydrogenases (GAPDHs; GapC1 and GapC2) undergo inhibitory *S*-nitrosylation and *S*-glutathionylation at Cys150, whereas H_2_S-induced persulfidation enhances GAPDH activity. Specifically, persulfidation at Cys160 promotes both catalytic activation and cytosol-to-nucleus translocation, linking metabolism to nuclear signaling (Zaffagnini *et al*., 2012; Aroca *et al*., 2015, 2017). Similarly, persulfidation of cytosolic glucose-6-phosphate dehydrogenase (G6PDH) in Arabidopsis (Cys159) and tomato (Cys155) modifies oligomeric structure, increases NADP^+^ affinity, and enhances activity, especially under salt stress (Wang *et al*., 2023). Persulfidation of succinate dehydrogenase (SDH, Complex II) stimulates mitochondrial respiration and ATP production, supporting guard cell energetics and stomatal regulation (Zhang *et al*., 2025). Additionally, persulfidation activates the H_2_S-producing enzyme DES1 (Cys44, Cys205), increasing intracellular sulfide levels and contributing to downstream stress signaling (Shen *et al*., 2020).

Persulfidation also fine-tunes antioxidant systems that maintain cellular redox homeostasis. Cytosolic ascorbate peroxidase (APX1) in Arabidopsis is activated by persulfidation (Aroca *et al*., 2015; Wang *et al*., 2021). In tomato, specific modifications of APX1 (Cys234) and the class III peroxidase POD5 (Cys61) enhance their activities, whereas persulfidation of catalase (CAT1, Cys234) decreases activity. These differential effects suggest that this PTM dynamically redistributes antioxidant capacity to optimize ROS detoxification during stress (J. Li *et al*., 2020).

Autophagy-related proteins represent another important persulfidation module, linking cellular recycling to stress adaptation. During endoplasmic reticulum (ER) stress, persulfidation of ATG18a at Cys103 enhances lipid binding and inhibits autophagosome maturation, while abscisic acid (ABA)-induced modification of AtATG4a at Cys170 suppresses its protease activity and modulates autophagic flux (Laureano-Marín *et al*., 2020; Aroca *et al*., 2021). Persulfidation additionally regulates diverse stress proteins: modification of the BOI-related E3 ubiquitin ligase BRG3 alters ubiquitination and transcription factor interactions, and persulfidation of the plasma membrane H^+^-ATPase PMA1 promotes ion homeostasis and salt tolerance (Ma *et al*., 2023; Sun *et al*., 2023).

A prominent hub of persulfidation-dependent regulation is ABA signaling. A well-characterized example is the regulation of ABA responses in guard cells, in which H_2_S modulates stomatal movements through persulfidation of key components such as SnRK2.6, DES1, and RBOHD (Chen *et al*., 2020; Shen *et al*., 2020). H_2_S enhances ROS production through persulfidation of the NADPH oxidase RBOHD at Cys825 and Cys890, promoting ABA-induced stomatal closure (Shen *et al*., 2020). Concurrently, persulfidation of the protein kinase SnRK2.6 at Cys131 and Cys137 increases kinase activity, improves phosphate transfer efficiency at Ser175, and strengthens interaction with the transcription factor ABF2, synergistically amplifying ABA and drought responses (Chen *et al*., 2020, 2021). At the transcriptional level, persulfidation of ABI4 at Cys250 enhances its stability and regulatory activity, linking ABA signaling with mitogen-activated protein kinase (MAPK) cascades and controlling seed germination. Additional ABA-associated transcription factors, including WRKY and basic helix–loop–helix (bHLH) proteins, undergo persulfidation that modulates DNA binding and protein interactions, contributing to stress adaptation and developmental regulation, such as delayed fruit ripening (Liu *et al*., 2019; Zhou *et al*., 2021; Sun *et al*., 2023).

Beyond ABA, persulfidation shapes wider stress signaling pathways. Persulfidation of MPK4, probably occurring on multiple Cys residues, enhances kinase activity and supports cold stress tolerance (Du *et al*., 2021). More generally, persulfidation of transcription factors integrates hormonal and environmental signals by modulating DNA binding, protein stability, and protein–protein interactions. Crosstalk with NO-dependent Cys modifications adds an additional regulatory layer that refines stress signaling outputs (Aroca *et al*., 2015, 2017, 2018).

### Connections between cysteine oxidation, reactive oxygen species, and persulfidation

A growing body of evidence supports a mechanistic coupling between Cys oxidation and persulfidation in plants (Filipovic *et al*., 2018; Zivanovic *et al*., 2019). Proteomic comparisons in *Arabidopsis thaliana* show that ∼5% of the proteome is persulfidated and that hundreds of proteins overlap with the sulfenylome, particularly proteins involved in stress responses, redox regulation, photosynthesis, and central metabolism (Zhou *et al*., 2020; Romero *et al*., 2026). Many proteins undergo both sulfenylation and persulfidation during oxidative stress, supporting the concept that ROS-mediated Cys oxidation primes proteins for persulfide formation (Filipovic *et al*., 2018; Zhou *et al*., 2020). This relationship is especially evident in photorespiration: when cellular H_2_O_2_ levels rise and the photorespiratory flux is suppressed, persulfidation of photorespiratory enzymes increases while sulfenylation decreases, highlighting a dynamic ROS–RSS interplay that stabilizes redox homeostasis (Aroca *et al*., 2023).

Functional studies across multiple plant pathways reinforce this oxidative priming model. In kinase signaling, oxidation of MPK4 at Cys181 is required for kinase activation and facilitates subsequent persulfidation, contributing to cold stress tolerance (Du *et al*., 2021). Similarly, reversible oxidation of cytosolic G6PDH under salt stress promotes persulfidation and protects the enzyme from irreversible overoxidation, preserving NADPH production required for antioxidant defense (Wang *et al*., 2023). Comparable mechanisms operate in glycolysis, where GAPDH undergoes cycles of oxidation, *S*-nitrosylation, and persulfidation that coordinate metabolic flux with cellular redox status (Bedhomme *et al*., 2012; Zaffagnini *et al*., 2013; Aroca *et al*., 2015).

Antioxidant enzymes provide additional plant-specific examples of ROS–persulfidation coupling. APX1, catalase, and class III peroxidases exhibit Cys modifications that alter activity in response to oxidative stress (Aroca *et al*., 2017; J. Li *et al*., 2020). H_2_S-mediated persulfidation can enhance APX and peroxidase activity while differentially modulating catalase function, enabling rapid adjustment of antioxidant capacity and preventing irreversible oxidative damage (J. Li *et al*., 2020). Likewise, NADPH oxidases such as RBOHD, which are themselves ROS producers, are regulated by persulfidation, creating feedback loops that integrate ROS generation with sulfur signaling (Shen *et al*., 2020).

Redox-dependent Cys switches also regulate transcription in plants. *S*-nitrosylation and persulfidation modify bHLH, WRKY, and other transcription factors. These changes affect DNA binding, protein stability, and promoter selection, which shape stress-responsive gene expression (Aroca *et al*., 2017; Liu *et al*., 2019). These reversible Cys modifications allow plants to quickly adjust transcriptional networks during abiotic stress and hormonal signaling (Aroca *et al*., 2017, 2018). Similar mechanisms are found in mammals. For example, GAPDH and NF-κB p65 are persulfidated at redox-sensitive Cys residues under oxidative or nitrosative stress, showing that ROS–RSS crosstalk is evolutionarily conserved (Filipovic *et al*., 2018; Zivanovic *et al*., 2019).

## Involvement of hydrogen sulfide in the responses to hypoxia

### Hydrogen sulfide participates in O_2_-dependent responses in animal cells

In mammalian cells, O_2_ sensing is achieved by (de)stabilization of the dimeric transcription factor Hypoxia-Inducible Factor (HIF) which is dependent on O_2_ availability (Ivan *et al*., 2001). Under normoxic conditions, one or both regulatory prolyl residues in HIF-α are hydroxylated by Prolyl Hydroxylase Domain (PHD) enzymes. The hydroxylated protein is recognized by the von Hippel–Lindau protein (pVHL) E3 ligase complex and targeted for proteasomal degradation. PHDs behave as O_2_-sensing enzymes: physiological levels of hypoxia indeed inhibit PHD activity, allowing HIF-α to accumulate, dimerize with HIF-β, and activate transcription of genes involved in anaerobic metabolism, angiogenesis, erythropoiesis, and cell survival (Kaelin and Ratcliffe, 2008).

The first demonstration of other gaseous molecules involved in the modulation of the HIF/VHL pathway concerned NO (Berchner-Pfannschmidt *et al*., 2010). Early studies showed that high concentrations of NO can induce HIF-1α stability, probably by inhibiting the activity of PHDs, either by competition with O_2_ at the ferrous iron (Fe^2+^) in the catalytic site of the enzymes or by reacting with Cys residues within the protein (Metzen *et al*., 2003; Chowdhury *et al*., 2011). More recently, a further mechanism of indirect regulation of O_2_ sensing by NO has been identified at the level of 2-aminoethanethiol dioxygenase (ADO), a second O_2_-sensing enzyme of humans with functional homology to oxygen-sensing plant cysteine oxidase (PCO) enzymes (Masson *et al*., 2019). NO can restrain ADO activity by affecting O_2_ availability through inhibition of Complex IV, ultimately altering accumulation levels of ADO targets, such as the G-protein signaling protein 4 (RGS4) (Kim *et al*., 2025).

Increasing evidence also indicates that H_2_S can modulate the HIF/pVHL O_2_-sensing pathway (Fig. 3) (Peng *et al*., 2010; Olson, 2015; Wu *et al*., 2015). PHD2, the prominent PHD isoform in humans, contains two Cys residues that are reported to undergo persulfidation (Dey *et al*., 2020) at Cys21 and Cys33 (Dey *et al*., 2020); this persulfidation is essential for PHD2 association with HIF-1α and consequent hydroxylation. H_2_S synthesis through CBS therefore promotes PHD2 activity and enhances HIF-1α hydroxylation, making it more susceptible to pVHL-mediated degradation. Notably, CBS expression was reported to decrease under hypoxia, suggesting that lower endogenous H_2_S production may contribute to HIF-1α stabilization during O_2_ deprivation (Dey *et al*., 2020). Thereby, CBS assumes an active role in monitoring O_2_-deficiency responses in humans. However, the effects of H_2_S on the hypoxic responses appear to be multifaceted. For example, exposure of Hep3B cells to 100 μM NaHS (an H_2_S donor) has been shown to induce HIF-1α degradation under 1% O_2_, but not under anoxia (0% O_2_), indicating that O_2_ may interact with H_2_S, influencing its effect on HIF-1α (Kai *et al*., 2012). Additionally, H_2_S can influence HIF-1α at the translational level. It has been demonstrated that exogenous NaHS suppresses hypoxia-induced HIF-1α accumulation by enhancing phosphorylation of eukaryotic translation initiation factor 2α (eIF2α), resulting in reduced HIF-1α translation and decreased expression of downstream targets such as the vascular endothelial growth factor (VEGF) (Wu *et al*., 2012). This pVHL-independent inhibition reveals an additional downstream layer of control through which H_2_S may modulate hypoxic signaling. Such conflicting observations may be attributed to differences in cell type, H_2_S concentration, redox state, and experimental conditions (Budde and Roth, 2010; Lohninger *et al*., 2015; Dey *et al*., 2020).

**Fig. 3.**
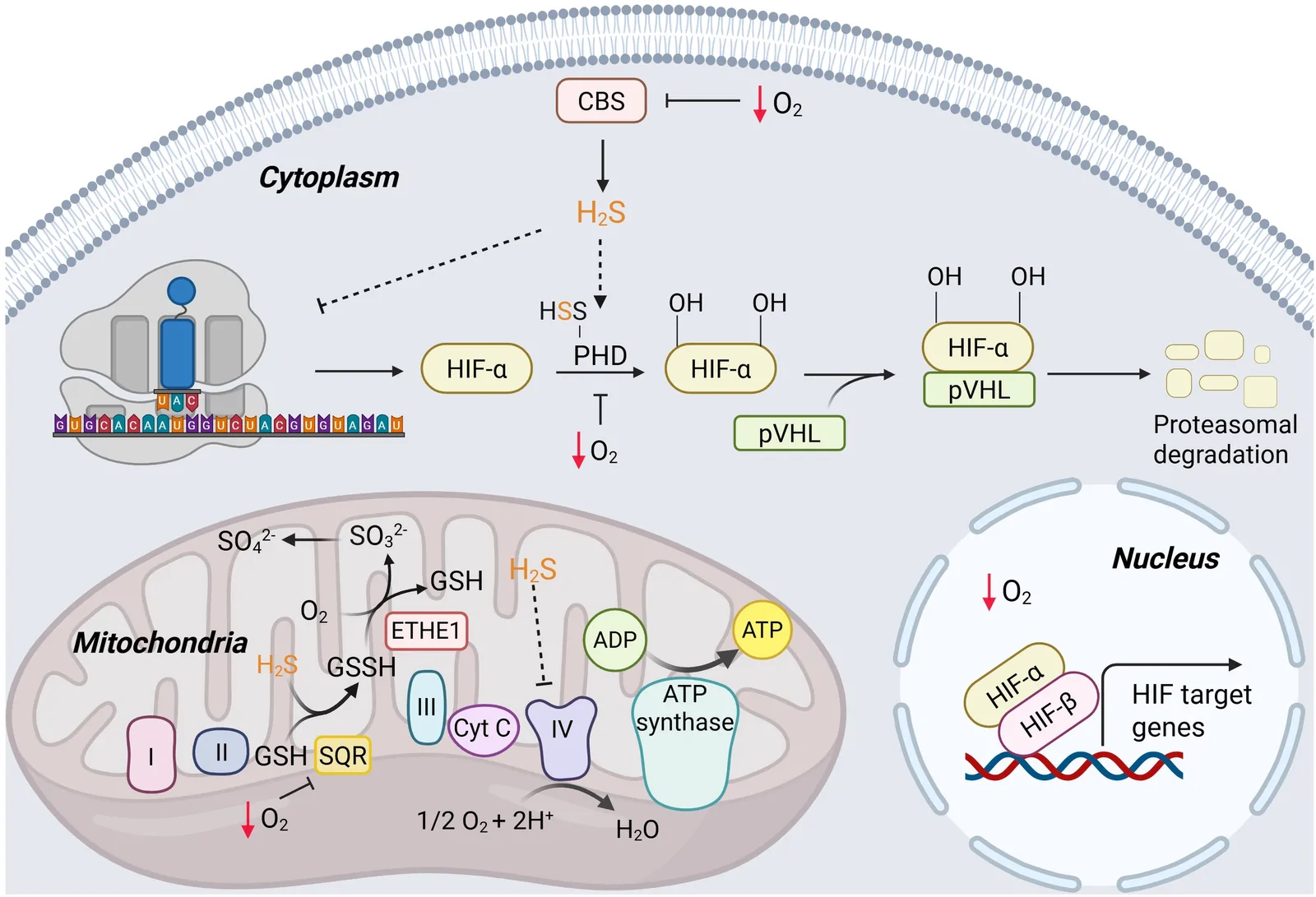
Effects of H_2_S on O_2_ sensing in mammalian cells. Dotted lines represent the proposed effects of H_2_S on O_2_-related proteins and processes. H_2_S produced by the CBS pathway can modulate the activity of HIF-α, both at the translational level and by increasing its degradation through enhancement of PHD activity via persulfidation. In addition, indirect repression of HIF-α activity may be associated with a modulation of mitochondrial O_2_ levels, following the inhibition of Complex IV by H_2_S. HIF-α stabilization and association with the HIF-β subunit promotes nuclear transcription under hypoxia. CBS, cystathionine β-synthase; HIF, hypoxia-inducible factor; pVHL, Von Hippel–Lindau tumor suppressor; PHD, prolyl-hydroxylase domain; SQR, sulfide:quinone oxidoreductase; ETHE1, ethylmalonic encephalopathy protein 1. Created in BioRender. Triozzi, P. (2026) https://BioRender.com/lr4cxu9.

Beyond mammalian cell culture systems, a role for H_2_S in O_2_ sensing is supported by evidence from multiple model organisms. In zebrafish embryos, silencing of CBS generates a hypoxia-like phenotype, which is recovered by exogenous NaHS supplementation, suggesting that endogenous H_2_S contributes to low O_2_ tolerance (Dey *et al*., 2020). In *Caenorhabditis elegans*, H_2_S promotes stabilization of HIF-1α through a mechanism independent of pVHL, indicating a further unknown mechanism whereby H_2_S intersects with O_2_-sensing pathways, possibly through modulation of other redox-sensitive proteins (Budde and Roth, 2010).

H_2_S can be produced and consumed by mitochondria, and its catabolism requires O_2_ via the sulfide-oxidizing pathway, which involves enzymes such as sulfide:quinone oxidoreductase (SQR) (Jackson *et al*., 2012), ethylmalonic encephalopathy protein 1 (ETHE1) (Tiranti *et al*., 2009), and sulfite oxidase (Fu *et al*., 2025). Therefore, low O_2_ conditions can limit mitochondrial H_2_S oxidation, resulting in elevated intracellular levels (Marutani *et al*., 2021; Paul *et al*., 2021) (Fig. 3). H_2_S accumulation can in turn protect cells by acting as a negative regulator of cytochrome *c* oxidase (Complex IV), thereby lowering O_2_ consumption, reducing mitochondrial membrane potential, and decreasing ROS formation (Olson, 2015). These effects may support mitochondrial functions during O_2_ scarcity by limiting energy demand and preventing oxidative damage. However, excessive H_2_S accumulation, for instance due to deficiency in SQR or ETHE1 activity, results in the irreversible inhibition of Complex IV, which can lead to energetic deficiency even in the presence of O_2_ (Tiranti *et al*., 2009). On the contrary, SQR expression improves mitochondrial respiration, maintains ATP production during hypoxia, and prevents sulfide-induced respiratory collapse. These findings suggest that the balance between H_2_S production and oxidation is a key determinant of cellular adaptation to low O_2_ levels (Marutani *et al*., 2021).

In adult mammals, the importance of H_2_S in physiological O_2_ sensing is most evident in the carotid bodies, small sensory organs that monitor arterial O_2_ and regulate ventilation (Kumar and Prabhakar, 2012). Double knockout mice for CSE fail to increase H_2_S levels in the carotid body during low O_2_ exposure and exhibit severely impaired ventilatory responses; in addition, pharmacological inhibition of CSE produces similar effects (Peng *et al*., 2010). These findings indicate that CSE-derived H_2_S is essential for the correct O_2_ sensing responses.

### Hydrogen sulfide in plant low O_2_ responses

In plants, appropriate biosynthesis of H_2_S underlies effective responses to low O_2_ stresses, as demonstrated by lower submergence tolerance of the Arabidopsis H_2_S-defective mutant *des1* (Zhou *et al*., 2021; Telara *et al*., 2025, Preprint). However, a full understanding of the endogenous H_2_S dynamics in tissues of plants exposed to low O_2_ is still lacking. In waterlogged roots of *Prunus persica* (Xiao *et al*., 2020) and root tips of *Pisum sativum* (Cheng *et al*., 2013), as well as in root tips from submerged *Zea mays* seedlings (Peng *et al*., 2016), H_2_S has been found to increase slightly as compared with control tissues after long-lasting treatments (24–72, 12–36, and 24–36 h, respectively). Submerged leaves of Arabidopsis can transiently accumulate H_2_S in the first hours of stress, returning to basal levels after 12 h, according to two distinct studies where a comparable treatment set-up was applied (Yang *et al*., 2021; Xuan *et al*., 2022). However, no H_2_S accumulation has been reported in Arabidopsis seedlings treated for a few hours with gaseous hypoxia (Telara *et al*., 2025, Preprint) or anoxia (Zhou *et al*., 2021), suggesting that physical entrapment of gaseous molecules is important to enable H_2_S build-up. As stated before, most intracellular sulfur is not present as freely diffusible H_2_S gas (its p*K*a being close to 7), but rather as hydrosulfide ion subjected to the acid trap mechanism. Nonetheless, lower diffusion of the volatile fraction may still contribute to the overall H_2_S balance in submerged tissues.

H_2_S accumulation under stress is generally accompanied by elevated expression of cysteine desulfhydrase genes (Fig. 1). While this is well documented in several stresses, including drought, salinity, cold, oxidative stress, and ABA treatment, as well as during bacterial pathogen infection (Vojtovič *et al*., 2021), few studies have investigated the transcriptional regulation of H_2_S biosynthetic genes under low oxygen and, surprisingly, none has revealed *DES1* up-regulation (Yang *et al.*, 2021; Xuan *et al*., 2022). H_2_S steady state is the result of biosynthesis and consumption, due to H_2_S incorporation into Cys by the action of cysteine synthase complexes, reactions with oxidized sulfur compounds, and formation of persulfide groups in proteins (Romero *et al*., 2014; Aroca *et al*., 2018; Filipovic *et al*., 2018). Lack of SQR homologous sequences in Arabidopsis (Theissen *et al*., 2003) makes the occurrence of the canonical mitochondrial sulfide oxidation pathway found in animals (Fig. 3) uncertain. The block of mitochondrial sulfide oxidation, serving as a main mechanism for H_2_S induction under hypoxia in animals, is likely to be replaced in plants by the regulation of other processes in sulfur metabolism (sulfate assimilation, GSH metabolism, mitochondrial sulfur catabolism under starvation through ETHE1, and Cys synthesis) (Krüßel *et al*., 2014) that alter H_2_S sinks and ultimately its steady state under low oxygen. Differences in global persulfidation might also account for the discrepancy observed between hypoxic seedlings (Telara *et al*., 2025, Preprint), where H_2_S levels decreased, and those treated with anoxia by Zhou *et al*. (2021), where no decrease was reported. Both conditions generate ROS (Pucciariello and Perata, 2017), but anoxia may elicit a different ROS production profile, determining lower availability of oxidized protein targets for H_2_S interaction than hypoxia, and thereby resulting in a weaker H_2_S sink. Higher ROS generation in anoxia is indeed likely to push protein thiol oxidation to Cys sulfinic/sulfonic acid, rather than to the Cys sulfenic state that can support persulfidation.

Discrepancies in H_2_S content recorded in the literature might also be reflecting tissue-specific dynamics in H_2_S regulation, making the comparison of different experimental designs misleading. For example, Zhou *et al*. (2021) analyzed free H_2_S in the roots of *A. thaliana*, whereas Telara *et al*. (2025, Preprint) focused on the whole seedlings. H_2_S content has been mainly determined in whole tissue extracts, using the methylene blue method (Cheng *et al*., 2013; Xiao *et al*., 2020; Yang *et al*., 2021; Xuan *et al*., 2022), or whole seedlings (via GC-MS/MS; Telara *et al*., 2025, Preprint); occasionally, it has been revealed by *in situ* detection of the H_2_S-reactive fluorescent probe WSP-1 (Peng *et al*., 2016; Yang *et al*., 2021; Zhou *et al*., 2021). Techniques characterized by higher spatial resolution (such as the use of microelectrodes or genetically encoded reporters for H_2_S) may further disclose the existence of tissue-specific regulation of H_2_S production under stress, which is presently unknown.

The possibility of modulating plant responses to low O_2_ stresses by acting on their H_2_S levels has been explored in Arabidopsis and a few other species, where evidence has been gathered that pre-treatments with the H_2_S donor NaHS can protect plants against subsequent submergence or waterlogging stress (Table 1). H_2_S appears to be implicated in plant low O_2_ physiology at different levels, by regulating relevant signaling, stress response, and metabolic processes (Fig. 4A). The activation of antioxidant defenses appears to be a common mechanism underlying the beneficial effects of H_2_S on plant survival to different conditions, ranging from heavy metal poisoning, temperature stress, salinity, to drought (reviewed in Zhou *et al*., 2025). This has been shown to occur through the regulation of either antioxidant gene expression or the levels of redox metabolites (Birke *et al*., 2015), or through persulfidation of relevant stress-associated enzymes (Aroca *et al*., 2015). However, a description of stress-specific mechanisms is generally not available. Under submergence or waterlogging, NaHS pre-treatments enhance the activity of ROS-scavenging enzymes such as superoxide dismutase, catalase, and APX, GSH synthesis, and the activity of alternative oxidase in mitochondria (Cheng *et al*., 2013; Peng *et al*., 2016; Xiao *et al*., 2020; Zhong *et al*., 2023). The increased antioxidant capacity can in turn alleviate the stress-induced death of root tips, a ROS-sensitive tissue.

**Fig. 4.**
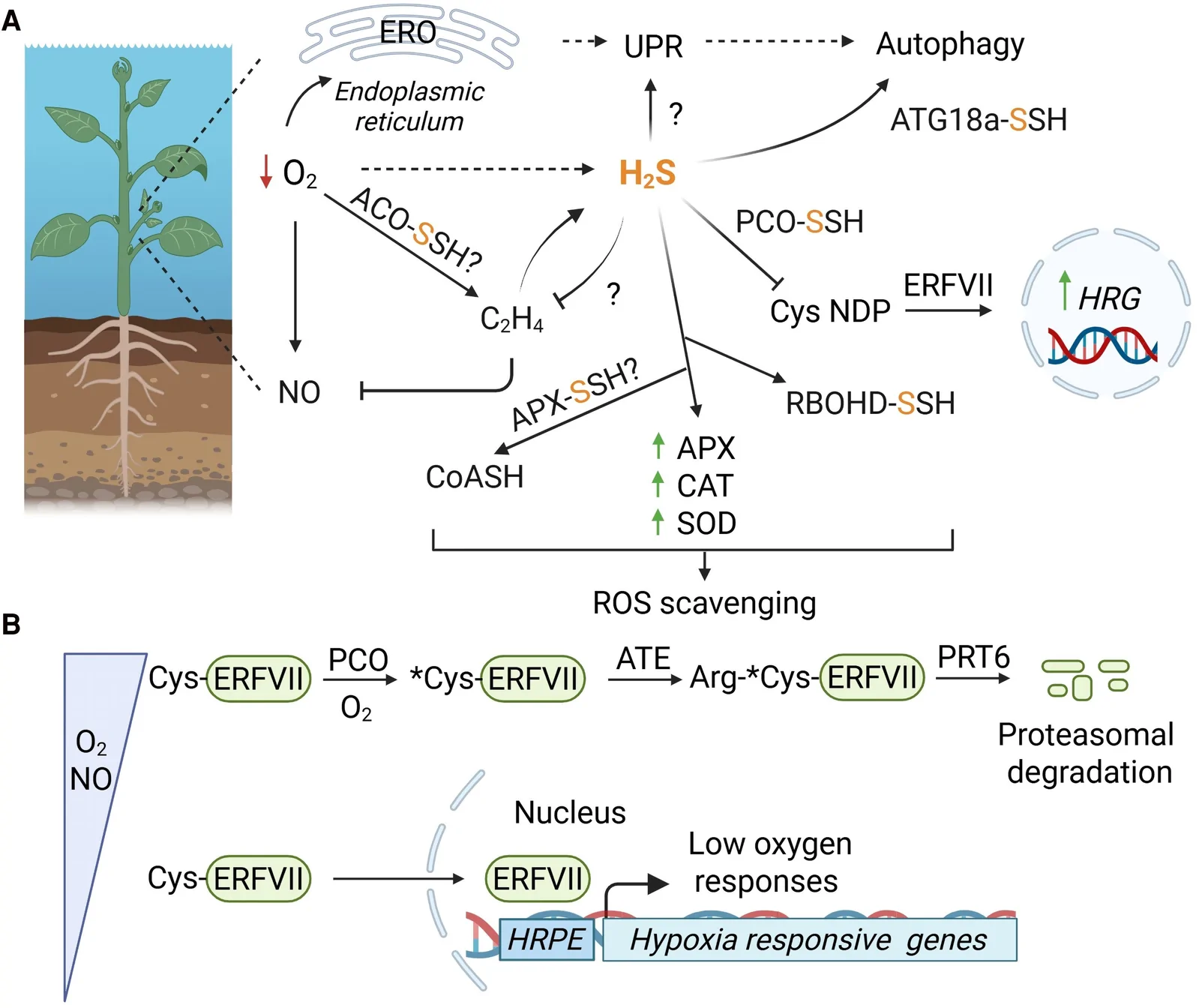
Involvement of H_2_S as a gasotransmitter during flooding-associated stresses. (A) Summary of mechanisms of H_2_S signaling under submergence or waterlogging. Proposed regulatory roles include ROS scavenging, crosstalk with hormone pathways, interactions with autophagy, and the modulation of PCO-dependent O_2_ sensing through protein persulfidation (-SSH). Dotted lines represent multistep or indirect processes. Hypothetical mechanisms are indicated by question marks. (B) The cysteine N-degron pathway (Cys-NDP) mediates the conditional degradation of the ERFVII transcription factors in the presence of oxygen and nitric oxide (above) via the sequential action of PCO, ATE, and PRT6 enzymes. Hypoxia prevents PCO action, thereby stabilizing the ERFVII factors and enabling hypoxic gene expression (below). *Cys, oxidized cysteine; APX, ascorbate peroxidase (*AT1G07890*); ATG18a, autophagy-related protein 18a (*AT3G24220*); ATE, arginyl-tRNA protein transferase (*ATE1*: *AT5G05700*; *ATE2*: *AT5G63280*); CAT, catalase (*AT4G35090*); CoASH, coenzyme A; ERFVII, ethylene response factor VII (*RAP2*.12: *AT1G53910*, *RAP2*.2: *AT3G14230*, *RAP2*.3: *AT3G16770*, *HRE1*: *AT1G72360*, *HRE2*: *AT2G40340*); ERO, endoplasmic reticulum oxidoreductin (*ERO1*: *AT1G15100*, *ERO2*: *AT2G25660*); HRG, hypoxia-responsive genes; HRPE, hypoxia-responsive promoter element; PCO, plant cysteine oxidase (*PCO1–PCO5*: *AT5G15120*, *AT1G05160*, *AT3G03040*, *AT4G36940*, *AT2G44810*); PRT6, E3 ubiquitin ligase proteolysis 6 (*AT5G02310*); RBOHD, respiratory burst oxidase homolog D (*AT5G47910*); SOD, superoxide dismutase (*AT1G08830*). Created in BioRender. Giuntoli, B. (2026) https://BioRender.com/yupkfkj.

**Table 1.** Overview of the impact of exogenous NaHS treatments on plant responses to low O_2_ stresses

Low oxygen stress	H_2_S treatment	Effect on stress response	Growth stage	Species	Reference
Dark submergence	Pre-treatment performed by 1 h 100 µM NaHS misting every 12 h for 3 d	Increased survival rate	Adult plants	*A. thaliana*	Yang *et al*. (2021)
Dark submergence	Pre-treatment performed by 1 h 100 µM NaHS misting every 12 h for 3 d	Increased survival rate, increased membrane integrity leaves	Adult plants	*A. thaliana*	Xuan *et al*. (2022)
Dark submergence	Fumigation with 50 µM NaHS solution for 24 h before the stress	Decreased root tip death, decreased ROS accumulation	Seedlings	*A. thaliana*	Xuan *et al*. (2022)
Dark submergence	Pre-treatment with 100 µM NaHS spraying (1 ml) every 12 h for 1.5 d	Increased survival rate	Adult plants	*A. thaliana*	Zhou *et al*. (2021)
Waterlogging under photoperiod 15 h light/9 h dark	Pre-treatment performed by transfer to 20–600 µM NaHS Hoagland solution for 1 h every 12 h for 1.5 d (best effect at 100 µM)	Alleviation of hypoxia- and re-oxygenation-induced root tip death, lower ROS accumulation in root tips, decreased ethylene accumulation	Seedlings	*Pisum sativum*	Cheng *et al*. (2013)
Submergence under light condition	Pre-treatment performed by transfer to 20–1000 µM NaHS Hoagland solution for 1.5 h every 12 h for 2 d (best effect at 100 µM)	Alleviation of hypoxia-induced root tip death, stimulation of antioxidant systems	Seedlings	*Zea mays*	Peng *et al*. (2016)
Waterlogging under photoperiod 15 h light/9 h dark	20–300 µM NaHS supplementation in water at the onset of the stress (best effect at 200 µM)	Alleviation of hypoxia-induced root tip death, decreased ethylene accumulation in roots, decreased ROS accumulation in roots and leaves	Seedlings	*Prunus persica*	Xiao *et al*. (2020)
Waterlogging under 12 h of light per day	10–1000 µM (best effect at 100 µM and 200 µM)	Decreased ROS accumulation, induction of Ca^2+^ influx, decreased mitochondrial damage, activation of alternative respiratory pathways in roots	Seedlings	*Avicennia marina*	Zhong *et al*. (2023)

Submergence refers to complete flooding of the plants, and waterlogging to root flooding.

The involvement of H_2_S in the regulation of autophagic processes appears to extend to submergence conditions. Autophagy is essential to plant survival under submergence (Chen *et al*., 2015; Guan *et al*., 2019; Yang *et al*., 2025), and its promotion has been indicated as a mechanism co-occurring with the improved submergence tolerance of H_2_S-pre-treated plants (Xuan *et al*., 2022). Therefore, while sulfide represses autophagy triggered by carbon starvation in normoxic conditions (Álvarez *et al*., 2012b; Aroca and Gotor, 2022), it instead exerts pro-autophagy effects in submergence conditions, suggesting that, under low O_2_ conditions, H_2_S signaling interacts with distinct mechanisms of carbon management. Additional mechanisms might involve interactions between H_2_S, autophagy, and low O_2_-induced ER stress. Different stressors, including hypoxia, can cause the accumulation of misfolded proteins in the ER, leading to the onset of ER stress conditions (Pereira *et al*., 2014; Bartoszewska and Collawn, 2020; Ugalde *et al*., 2022) and the activation of protective unfolded protein response (UPR) pathways. In mammals, the involvement of H_2_S in the UPR has been reported (Krishnan *et al*., 2011). Remarkably, in plants, the UPR is regulated by O_2_-dependent endoplasmic reticulum oxidoreductin (ERO) 1/2 enzymes, whose mutation in Arabidopsis impairs survival upon low O_2_ (Zhou *et al*., 2021; Ugalde *et al*., 2022). On the other hand, in normoxic conditions, ER stresses can trigger ER degradation through autophagy (also called ER-phagy), with the participation of H_2_S signaling acting through ATG18a persulfidation (Aroca *et al*., 2021). While mechanistic evidence is missing, these separate elements suggest that H_2_S signaling might be connected with hypoxia-activated UPR pathways and ER-phagy in plants (Fig. 4A).

Pre-treatment with NaHS causes changes in amino acid metabolism, hormone production, and signal transduction taking place during subsequent submergence. The interplay between H_2_S and hormone regulation under low O_2_ has not been extensively characterized. Evidence of repression of cytokinin- and auxin-dependent signaling emerges from transcriptomic data in Arabidopsis (Yang *et al*., 2021). Moreover, it has been proposed that H_2_S might improve plant defense and antioxidant responses in the desubmergence phase through the activation of jasmonic acid-dependent signaling (Yuan *et al*., 2017; Yang *et al*., 2021). Interestingly, H_2_S has been found to participate in a negative feedback regulation with ethylene, the major flooding-related hormone. H_2_S can act downstream of ethylene during stress responses, mediating part of the protective effects of ethylene, such as in the case of ethylene-induced stomatal closure, while also down-regulating ethylene biosynthesis (Liu *et al*., 2012; Hou *et al*., 2013; Jia *et al*., 2018). Also in waterlogged roots, H_2_S pre-treatment has been found to counteract ethylene accumulation (Cheng *et al*., 2013; Xiao *et al*., 2020). Although vastly implied by the literature (Liu *et al*., 2021), the interactions between H_2_S and hormone-dependent physiological phenomena are supported by surprisingly few mechanistic links. These encompass persulfidation of SnRK2.6 and RBOHD in guard cells (Chen *et al*., 2020; Shen *et al*., 2020), and of the ethylene biosynthetic enzyme 1-aminocyclopropane-1-carboxylic acid (ACC) oxidase of *Solanum lycopersicum* during exposure to osmotic stress (Jia *et al*., 2018). The latter modification may underlie ethylene down-regulation during waterlogging and, potentially, regulate stomatal closure responses in this condition. The network of gaseous signals that crosstalk under low O_2_ conditions further extends to NO. Ethylene, which is known to modulate NO levels under hypoxia through phytoglobins (Hartman *et al*., 2019), might interfere with downstream H_2_S signaling indirectly through NO–H_2_S synergy.

Chemical interventions with H_2_S donors and the use of *des1* mutants indicate that H_2_S is essential to the activation of hypoxia-inducible genes following the onset of low O_2_ conditions. In Arabidopsis, NaHS supplementation boosts hypoxia marker gene expression, whereas defective production of endogenous H_2_S associates with lower gene induction (Yang *et al*., 2021; Xuan *et al*., 2022; Telara *et al*., 2025, Preprint). Hypoxic transcription is coordinated by ERFVII (Ethylene Responsive Factor group VII) family transcription factors, which activate the expression of hypoxia-responsive genes by direct association with conserved *cis*-elements in their promoters (Gasch *et al*., 2016) (Fig. 4B). In turn, the ERFVII proteins are directly regulated by O_2_ availability through a mechanism of conditional destabilization similar to that impinging on HIF-1α in humans. In the presence of O_2_, PCO enzymes oxidize the conserved ERFVII residue Cys2, thereby funneling these factors into a dedicated pathway of proteasomal degradation, the Cys/Arg N-degron pathway (Weits *et al*., 2014). Conversely, lack of O_2_ inhibits PCOs and enables ERFVII accumulation, triggering the hypoxic response (Fig. 4B).

In this context, H_2_S has been shown to inhibit PCO activity in Arabidopsis, revealing that H_2_S participates in the induction of hypoxia-responsive genes by contributing to ERFVII stabilization (Telara *et al*., 2025, Preprint). Persulfidation has been detected on recombinant AtPCO4 upon H_2_S supplementation and, *in vivo*, in hypoxia-treated Arabidopsis seedlings (Telara *et al*., 2025, Preprint). Moreover, persulfidation of AtPCO3 has been reported by Aroca *et al*. (2017) in unstressed wild-type leaves. The occurrence of this PTM in the regulation of human and plant O_2_-sensing enzymes (namely PHDs and PCOs), although apparently deployed for opposite outcomes (Figs 3, 4), reinforces the relevance of H_2_S as a gasotransmitter under low O_2_. PCOs are also inhibited in the presence of H_2_O_2_ (Akter *et al*., 2026) and potentially affected by *S*-nitrosylation, as shown in the case of PCO3 (Hu *et al*., 2015). Besides their role as O_2_ sensors, PCOs thus appear to serve as a convergence point of ROS- and NO-mediated signaling pathways under hypoxia. It is tempting to speculate on the participation of H_2_S in the same mechanism, with PCO persulfidation occurring on catalytically relevant Cys residues previously targeted by oxidation or *S*-nitrosylation. Evidence for this scenario, and its implications for plant hypoxia physiology, will emerge from experiments aimed at disentangling the mechanisms of action of the signaling molecules involved in PCO enzyme regulation.

## Conclusions and perspectives

Historically regarded as a toxic metabolic byproduct, H_2_S has now been identified as the third gasotransmitter molecule in plants, after NO and CO, with wide-ranging beneficial effects. Recent studies have revealed its fundamental role in the regulation of plant development and the integration of plant defense responses against various abiotic stresses including drought, salinity, and flooding. Despite the heterogeneous experimental approaches adopted in the literature, an emerging consensus suggests that submergence tolerance is influenced by H_2_S levels. The beneficial effects of H_2_S have been traced back to its manyfold intersections with hypoxia-associated pathways of ROS management, ER signaling, autophagy, and O_2_ sensing. Moreover, H_2_S is now known to take part in signaling that involves the other two main gaseous signals implicated in plant hypoxic regulation, namely ethylene and NO.

H_2_S exerts its roles mainly through protein persulfidation. So far, direct hypoxic regulation of protein functions via persulfidation has only been demonstrated for PCO O_2_-sensing enzymes. PCO inhibition by H_2_S affects the stability of ERFVII transcription factors and the induction of hypoxia-responsive genes, ultimately influencing plant metabolic adaptations to low O_2_. However, hypoxia is likely to have a more widespread impact on protein persulfidation profiles, considering the effects of low O_2_ on NO levels and redox balance and their connections with H_2_S-dependent PTMs. Efforts aimed at the characterization of the persulfidome of plants exposed to O_2_ deprivation will provide relevant insights on the impact of H_2_S signaling under hypoxia.

The application of H_2_S to crops, potentially in combination with its synergistic partner NO, is considered as a viable protective strategy against abiotic stresses (Dos Reis *et al*., 2026). Crop priming with H_2_S-based formulations might be especially useful for high-value cereals such as barley or wheat, which suffer from severe yield losses due to flooding. Crop treatments where the active component is an endogenous gasotransmitter, rather than an ‘unnatural’ agrochemical, may be favorably adopted by farmers and consumers. The development of effective agricultural solutions derived from H_2_S administration is, however, still hindered by scant understanding of the tissue- and dose-dependent responses, in addition to known H_2_S dose-dependent toxicity effects. Although at low concentrations it plays a positive signaling role, at higher concentrations H_2_S can trigger toxicity by impairing mitochondrial function. Defining thresholds at which the transition from positive to negative function occurs in specific relevant crops is crucial to enable effective flooding protection strategies based on H_2_S application or crop genetic improvement. For the same reason, the choice of H_2_S donor compounds and the release kinetics of H_2_S from any formulation (Dos Reis *et al*., 2026) are expected to be crucial aspects in the development of H_2_S-based supplements for agriculture.

## References

[erag402-B1] Abe K, Kimura H. 1996. The possible role of hydrogen sulfide as an endogenous neuromodulator. The Journal of Neuroscience 16, 1066–1071. 10.1523/JNEUROSCI.16-03-01066.1996 PMC6578817 8558235

[erag402-B2] Akter S, Fu L, Jung Y, Lo Conte M, Reed Lawson J, Todd Lowther W, Sun R, Liu K, Yang J, Carroll KS. 2018. Chemical proteomics reveals new targets of cysteine sulfinic acid reductase. Nature Chemical Biology 14, 995–1004. 10.1038/s41589-018-0116-2 PMC6192846 30177848

[erag402-B3] Akter S, Perri M, Lavilla-Puerta M, et al 2026. H_2_O_2_ repurposes plant O_2_ sensing to regulate post-hypoxia responses. Nature 653, 1130–1138. 10.1038/s41586-026-10366-1 PMC13216066 42020755

[erag402-B4] Álvarez C, Calo L, Romero LC, García I, Gotor C. 2010. An *O*-acetylserine(thiol)lyase homolog with L-cysteine desulfhydrase activity regulates cysteine homeostasis in Arabidopsis. Plant Physiology 152, 656–669. 10.1104/pp.109.147975 PMC2815857 19955263

[erag402-B5] Álvarez C, Ángeles Bermúdez M, Romero LC, Gotor C, García I. 2012a. Cysteine homeostasis plays an essential role in plant immunity. New Phytologist 193, 165–177. 10.1111/j.1469-8137.2011.03889.x 21988475

[erag402-B6] Álvarez C, García I, Moreno I, Pérez-Pérez ME, Crespo JL, Romero LC, Gotor C. 2012b. Cysteine-generated sulfide in the cytosol negatively regulates autophagy and modulates the transcriptional profile in Arabidopsis. The Plant Cell 24, 4621–4634. 10.1105/tpc.112.105403 PMC3531856 23144183

[erag402-B7] Andrés CMC, Lobo F, de la Lastra JMP, Munguira EB, Juan CA, Pérez Lebeña E. 2025. Reactive sulfur species and protein persulfidation: an emerging redox axis in human health and disease. Current Issues in Molecular Biology 47, 765. 10.3390/cimb47090765 PMC12468119 41020887

[erag402-B8] Aroca A, Serna A, Gotor C, Romero LC. 2015. S-sulfhydration: a cysteine posttranslational modification in plant systems. Plant Physiology 168, 334–342. 10.1104/pp.15.00009 PMC4424021 25810097

[erag402-B9] Aroca A, Benito JM, Gotor C, Romero LC. 2017. Persulfidation proteome reveals the regulation of protein function by hydrogen sulfide in diverse biological processes in Arabidopsis. Journal of Experimental Botany 68, 4915–4927. 10.1093/jxb/erx294 PMC5853657 28992305

[erag402-B10] Aroca A, Gotor C, Romero LC. 2018. Hydrogen sulfide signaling in plants: emerging roles of protein persulfidation. Frontiers in Plant Science 9, 1369. 10.3389/fpls.2018.01369 PMC6157319 30283480

[erag402-B11] Aroca A, Gotor C, Bassham DC, Romero LC. 2020. Hydrogen sulfide: from a toxic molecule to a key molecule of cell life. Antioxidants 9, 621. 10.3390/antiox9070621 PMC7402122 32679888

[erag402-B12] Aroca A, Yruela I, Gotor C, Bassham DC. 2021. Persulfidation of ATG18a regulates autophagy under ER stress in Arabidopsis. Proceedings of the National Academy of Sciences, USA 118, e2023604118. 10.1073/pnas.2023604118 PMC8157962 33975948

[erag402-B13] Aroca A, Gotor C. 2022. Hydrogen sulfide: a key role in autophagy regulation from plants to mammalians. Antioxidants 11, 327. 10.3390/antiox11020327 PMC8868472 35204209

[erag402-B14] Aroca A, García-Díaz I, García-Calderón M, Gotor C, Márquez AJ, Betti M. 2023. Photorespiration: regulation and new insights on the potential role of persulfidation. Journal of Experimental Botany 74, 6023–6039. 10.1093/jxb/erad291 PMC10575701 37486799

[erag402-B15] Bartoszewska S, Collawn JF. 2020. Unfolded protein response (UPR) integrated signaling networks determine cell fate during hypoxia. Cellular & Molecular Biology Letters 25, 18. 10.1186/s11658-020-00212-1 PMC7071609 32190062

[erag402-B16] Bedhomme M, Adamo M, Marchand CH, Couturier J, Rouhie N, Lemaire SD, Zaffagnini M, Trost P. 2012. Glutathionylation of cytosolic glyceraldehyde-3-phosphate dehydrogenase from the model plant *Arabidopsis thaliana* is reversed by both glutaredoxins and thioredoxins *in vitro*. The Biochemical Journal 445, 337–347. 10.1042/BJ20120505 22607208

[erag402-B17] Beedle AEM, Lynham S, Garcia-Manyes S. 2016. Protein S-sulfenylation is a fleeting molecular switch that regulates non-enzymatic oxidative folding. Nature Communications 7, 12490. 10.1038/ncomms12490 PMC4996944 27546612

[erag402-B18] Benchoam D, Cuevasanta E, Möller MN, Alvarez B. 2019. Hydrogen sulfide and persulfides oxidation by biologically relevant oxidizing species. Antioxidants 8, 48. 10.3390/antiox8020048 PMC6406583 30813248

[erag402-B19] Berchner-Pfannschmidt U, Tug S, Kirsch M, Fandrey J. 2010. Oxygen-sensing under the influence of nitric oxide. Cellular Signalling 22, 349–356. 10.1016/j.cellsig.2009.10.004 19861159

[erag402-B20] Birke H, De Kok LJ, Wirtz M, Hell R. 2015. The role of compartment-specific cysteine synthesis for sulfur homeostasis during H_2_S exposure in Arabidopsis. Plant & Cell Physiology 56, 358–367. 10.1093/pcp/pcu166 25416292

[erag402-B21] Biteau B, Labarre J, Toledano MB. 2003. ATP-dependent reduction of cysteine-sulphinic acid by *S. cerevisiae* sulphiredoxin. Nature 425, 980–984. 10.1038/nature02075 14586471

[erag402-B22] Budde MW, Roth MB. 2010. Hydrogen sulfide increases hypoxia-inducible factor-1 activity independently of von Hippel–Lindau tumor suppressor-1 in *C. elegans*. Molecular Biology of the Cell 21, 212–217. 10.1091/mbc.E09-03-0199 PMC2801715 19889840

[erag402-B23] Cao H, Liang Y, Zhang L, Liu Z, Liu D, Cao X, Deng X, Jin Z, Pei Y. 2022. AtPRMT5-mediated AtLCD methylation improves Cd^2+^ tolerance via increased H_2_S production in Arabidopsis. Plant Physiology 190, 2637–2650. 10.1093/plphys/kiac376 PMC9706440 35972421

[erag402-B24] Chen J, Zhou H, Xie Y. 2021. SnRK2.6 phosphorylation/persulfidation: where ABA and H_2_S signaling meet. Trends in Plant Science 26, 1207–1209. 10.1016/j.tplants.2021.08.005 34493443

[erag402-B25] Chen L, Liao B, Qi H, et al 2015. Autophagy contributes to regulation of the hypoxia response during submergence in *Arabidopsis thaliana*. Autophagy 11, 2233–2246. 10.1080/15548627.2015.1112483 PMC4835207 26566261

[erag402-B26] Chen S, Jia H, Wang X, Shi C, Wang X, Ma P, Wang J, Ren M, Li J. 2020. Hydrogen sulfide positively regulates abscisic acid signaling through persulfidation of SnRK2.6 in guard cells. Molecular Plant 13, 732–744. 10.1016/j.molp.2020.01.004 31958520

[erag402-B27] Cheng W, Zhang L, Jiao C, Su M, Yang T, Zhou L, Peng R, Wang R, Wang C. 2013. Hydrogen sulfide alleviates hypoxia-induced root tip death in *Pisum sativum*. Plant Physiology and Biochemistry 70, 278–286. 10.1016/j.plaphy.2013.05.042 23800663

[erag402-B28] Chowdhury R, Flashman E, Mecinović J, Kramer HB, Kessler BM, Frapart YM, Boucher JL, Clifton IJ, McDonough MA, Schofield CJ. 2011. Studies on the reaction of nitric oxide with the hypoxia-inducible factor prolyl hydroxylase domain 2 (EGLN1). Journal of Molecular Biology 410, 268–279. 10.1016/j.jmb.2011.04.075 21601578

[erag402-B29] Corpas FJ, González-Gordo S, Cañas A, Palma JM. 2019. Nitric oxide and hydrogen sulfide in plants: which comes first? Journal of Experimental Botany 70, 4391–4404. 10.1093/jxb/erz031 30715479

[erag402-B30] Cortese-Krott MM, Koning A, Kuhnle GGC, et al 2017. The reactive species interactome: evolutionary emergence, biological significance, and opportunities for redox metabolomics and personalized medicine. Antioxidants & Redox Signaling 27, 684–712. 10.1089/ars.2017.7083 PMC5576088 28398072

[erag402-B31] Cuevasanta E, Lange M, Bonanata J, Coitiño EL, Ferrer-Sueta G, Filipovic MR, Alvarez B. 2015. Reaction of hydrogen sulfide with disulfide and sulfenic acid to form the strongly nucleophilic persulfide. Journal of Biological Chemistry 290, 26866–26880. 10.1074/jbc.M115.672816 PMC4646399 26269587

[erag402-B32] Dey A, Prabhudesai S, Zhan Y, Rao G, Thirugnanam K, Hossen MN, Dwivedi SKD, Ramchandran R, Mukherjee P, Bhattacharya R. 2020. Cystathione β-synthase regulates HIF-1α stability through persulfidation of PHD2. Science Advances 6, eaaz8534. 10.1126/sciadv.aaz8534 PMC7458453 32937467

[erag402-B33] Dóka É, Pader I, Bíró A. 2016. A novel persulfide detection method reveals protein persulfide- and polysulfide-reducing functions of thioredoxin and glutathione systems. Science Advances 2, e1500968. 10.1126/sciadv.1500968 PMC4737208 26844296

[erag402-B34] Dos Reis RA, Seabra AB, Aragão CB, Halfeld M, Nunes RS, Rodriguez R, Benavides-Mendoza A, Rubilar O, Tortella GR. 2026. Nitric oxide and hydrogen sulfide crosstalk in plants: redox regulation, stress adaptation, and emerging applications. International Journal of Molecular Sciences 27, 4962. 10.3390/ijms27114962 PMC13256797 42278488

[erag402-B35] Du X, Jin Z, Liu Z, Liu D, Zhang L, Ma X, Yang G, Liu S, Guo Y, Pei Y. 2021. H_2_S persulfidated and increased kinase activity of MPK4 to response cold stress in Arabidopsis. Frontiers in Molecular Biosciences 8, 635470. 10.3389/fmolb.2021.635470 PMC7991836 33778005

[erag402-B36] Fang H, Liu Z, Jin Z, Zhang L, Liu D, Pei Y. 2016. An emphasis of hydrogen sulfide–cysteine cycle on enhancing the tolerance to chromium stress in Arabidopsis. Environmental Pollution 213, 870–877. 10.1016/j.envpol.2016.03.035 27038574

[erag402-B37] Fang H, Liu Z, Long Y, Liang Y, Jin Z, Zhang L, Liu D, Li H, Zhai J, Pei Y. 2017. The Ca^2+^/calmodulin2-binding transcription factor TGA3 elevates LCD expression and H_2_S production to bolster Cr^6+^ tolerance in Arabidopsis. The Plant Journal 91, 1038–1050. 10.1111/tpj.13627 28670772

[erag402-B38] Fang H, Yu Z, Xing K, Zhou L, Shao Y, Zhang X, Pei Y, Zhang L. 2023. Transcriptomic analysis reveals the functions of H_2_S as a gasotransmitter independently of Cys in *Arabidopsis*. Frontiers in Plant Science 14, 1184991. 10.3389/fpls.2023.1184991 PMC10272727 37332712

[erag402-B39] Fang T, Cao Z, Li J, Shen W, Huang L. 2014. Auxin-induced hydrogen sulfide generation is involved in lateral root formation in tomato. Plant Physiology and Biochemistry 76, 44–51. 10.1016/j.plaphy.2013.12.024 24463534

[erag402-B40] FAO . 2025. The impact of disasters on agriculture and food security 2025: digital solutions for reducing risks and impacts. Rome: FAO. doi: 10.4060/cd7185en.

[erag402-B41] Filipovic MR, Zivanovic J, Alvarez B, Banerjee R. 2018. Chemical biology of H_2_S signaling through persulfidation. Chemical Reviews 118, 1253–1337. 10.1021/acs.chemrev.7b00205 PMC6029264 29112440

[erag402-B42] Fu C-Y, Kohl JB, Liebsch F, et al 2025. Sulfite oxidase deficiency causes persulfidation loss and H_2_S release. Journal of Clinical Investigations 135, e181299. 10.1172/JCI181299 PMC12578401 41178722

[erag402-B43] Furne J, Saeed A, Levitt MD. 2008. Whole tissue hydrogen sulfide concentrations are orders of magnitude lower than presently accepted values. American Journal of Physiology: Regulatory, Integrative and Comparative Physiology 295, R1479–R1485. 10.1152/ajpregu.90566.2008 18799635

[erag402-B44] García-Mata C, Lamattina L. 2010. Hydrogen sulphide, a novel gasotransmitter involved in guard cell signalling. New Phytologist 188, 977–984. 10.1111/j.1469-8137.2010.03465.x 20831717

[erag402-B45] Gasch P, Fundinger M, Müller JT, Lee T, Bailey-Serres J, Mustropha A. 2016. Redundant ERF-VII transcription factors bind to an evolutionarily conserved cis-motif to regulate hypoxia-responsive gene expression in Arabidopsis. The Plant Cell 28, 160–180. 10.1105/tpc.15.00866 PMC4746684 26668304

[erag402-B46] Giles GI, Nasim MJ, Ali W, Jacob C. 2017. The reactive sulfur species concept: 15 years on. Antioxidants 6, 38. 10.3390/antiox6020038 PMC5488018 28545257

[erag402-B47] González-Gordo S, Palma JM, Corpas FJ. 2020. Appraisal of H_2_S metabolism in *Arabidopsis thaliana*: *in silico* analysis at the subcellular level. Plant Physiology and Biochemistry 155, 579–588. 10.1016/j.plaphy.2020.08.014 32846393

[erag402-B48] Gotor C, García I, Aroca A, Laureano-Marín AM, Arenas-Alfonseca L, Jurado-Flores A, Moreno I, Romero LC. 2019. Signaling by hydrogen sulfide and cyanide through post-translational modification. Journal of Experimental Botany 70, 4251–4265. 10.1093/jxb/erz225 31087094

[erag402-B49] Guan B, Lin Z, Liu D, Li C, Zhou Z, Mei F, Li J, Deng X. 2019. Effect of waterlogging-induced autophagy on programmed cell death in Arabidopsis roots. Frontiers in Plant Science 10, 468. 10.3389/fpls.2019.00468 PMC6470631 31031792

[erag402-B50] Hartman S, Liu Z, van Veen H, et al 2019. Ethylene-mediated nitric oxide depletion pre-adapts plants to hypoxia stress. Nature Communications 10, 4020. 10.1038/s41467-019-12045-4 PMC6728379 31488841

[erag402-B51] Hosoki R, Matsuki N, Kimura H. 1997. The possible role of hydrogen sulfide as an endogenous smooth muscle relaxant in synergy with nitric oxide. Biochemical and Biophysical Research Communications 237, 527–531. 10.1006/bbrc.1997.6878 9299397

[erag402-B52] Hou Z, Wang L, Liu J, Hou L, Liu X. 2013. Hydrogen sulfide regulates ethylene-induced stomatal closure in *Arabidopsis thaliana*. Journal of Integrative Plant Biology 55, 277–289. 10.1111/jipb.12004 23134300

[erag402-B53] Hu J, Huang X, Chen L, Sun X, Lu C, Zhang L, Wang Y, Zuo J. 2015. Site-specific nitrosoproteomic identification of endogenously S-nitrosylated proteins in Arabidopsis. Plant Physiology 167, 1731–1746. 10.1104/pp.15.00026 PMC4378176 25699590

[erag402-B54] Hu K-D, Zhang XY, Yao GF, et al 2020. A nuclear-localized cysteine desulfhydrase plays a role in fruit ripening in tomato. Horticulture Research 7, 211. 10.1038/s41438-020-00439-1 PMC7736880 33328464

[erag402-B55] Huang J, Willems P, Wei B, et al 2019. Mining for protein S-sulfenylation in Arabidopsis uncovers redox-sensitive sites. Proceedings of the National Academy of Sciences, USA 116, 21256–21261. 10.1073/pnas.1906768116 PMC6800386 31578252

[erag402-B56] Ida T, Sawa T, Ihara H, et al 2014. Reactive cysteine persulfides and S-polythiolation regulate oxidative stress and redox signaling. Proceedings of the National Academy of Sciences, USA 111, 7606–7611. 10.1073/pnas.1321232111 PMC4040604 24733942

[erag402-B57] Ignarro LJ, Buga GM, Wood KS, Byrns RE, Chaudhuri G. 1987. Endothelium-derived relaxing factor produced and released from artery and vein is nitric oxide. Proceedings of the National Academy of Sciences, USA 84, 9265–9269. 10.1073/pnas.84.24.9265 PMC299734 2827174

[erag402-B58] Ivan M, Kondo K, Yang H, Kim W, Valiando J, Ohh M, Salic A, Asara JM, Lane WS, Kaelin J. 2001. HIFα targeted for VHL-mediated destruction by proline hydroxylation: implications for O_2_ sensing. Science 292, 464–468. 10.1126/science.1059817 11292862

[erag402-B59] Jackson MR, Melideo SL, Jorns MS. 2012. Human sulfide:quinone oxidoreductase catalyzes the first step in hydrogen sulfide metabolism and produces a sulfane sulfur metabolite. Biochemistry 51, 6804–6815. 10.1021/bi300778t 22852582

[erag402-B60] Jensen B, Fago A. 2018. Reactions of ferric hemoglobin and myoglobin with hydrogen sulfide under physiological conditions. Journal of Inorganic Biochemistry 182, 133–140. 10.1016/j.jinorgbio.2018.02.007 29459272

[erag402-B61] Jia H, Wang X, Dou Y, Liu D, Si W, Fang H, Zhao C, Chen S, Xi J, Li J. 2016. Hydrogen sulfide–cysteine cycle system enhances cadmium tolerance through alleviating cadmium-induced oxidative stress and ion toxicity in Arabidopsis roots. Scientific Reports 6, 39702. 10.1038/srep39702 PMC5177925 28004782

[erag402-B62] Jia H, Chen S, Liu D, et al 2018. Ethylene-induced hydrogen sulfide negatively regulates ethylene biosynthesis by persulfidation of ACO in tomato under osmotic stress. Frontiers in Plant Science 9, 1517. 10.3389/fpls.2018.01517 PMC6199894 30386366

[erag402-B63] Kabil O, Motl N, Banerjee R. 2014. H_2_S and its role in redox signaling. Biochimica et Biophysica Acta 1844, 1355–1366. 10.1016/j.bbapap.2014.01.002 PMC4048824 24418393

[erag402-B64] Kaelin WG, Ratcliffe PJ. 2008. Oxygen sensing by metazoans: the central role of the HIF hydroxylase pathway. Molecular Cell 30, 393–402. 10.1016/j.molcel.2008.04.009 18498744

[erag402-B65] Kai S, Tanaka T, Daijo H, Harada H, Kishimoto S, Suzuki K, Takabuchi S, Takenaga K, Fukuda K, Hirota K. 2012. Hydrogen sulfide inhibits hypoxia- but not anoxia-induced hypoxia-inducible factor 1 activation in a von Hippel–Lindau- and mitochondria-dependent manner. Antioxidants & Redox Signaling 16, 203–216. 10.1089/ars.2011.3882 PMC3234659 22004513

[erag402-B66] Kim H, Tian Y-M, Ratcliffe PJ, Keeley TP. 2025. Nitric oxide promotes cysteine N-degron proteolysis through control of oxygen availability. Proceedings of the National Academy of Sciences, USA 122, e2501796122. 10.1073/pnas.2501796122 PMC12403002 40828020

[erag402-B67] Kolupaev YE, Karpets YV, Shkliarevskyi MA, Yastreb TO, Plohovska SH, Yemets АI, Blume YB. 2022. Gasotransmitters in plants: mechanisms of participation in adaptive responses. The Open Agriculture Journal 16, e2207050.

[erag402-B68] Krishnan N, Fu C, Pappin DJ, Tonks NK. 2011. H_2_S-induced sulfhydration of the phosphatase PTP1B and its role in the endoplasmic reticulum stress response. Science Signaling 4, ra86. 10.1126/scisignal.2002329 PMC3328411 22169477

[erag402-B69] Krüßel L, Junemann J, Wirtz M, et al 2014. The mitochondrial sulfur dioxygenase ETHYLMALONIC ENCEPHALOPATHY PROTEIN1 is required for amino acid catabolism during carbohydrate starvation and embryo development in Arabidopsis. Plant Physiology 165, 92–104. 10.1104/pp.114.239764 PMC4012607 24692429

[erag402-B70] Kumar P, Prabhakar NR. 2012. Peripheral chemoreceptors: function and plasticity of the carotid body. Comprehensive Physiology 2, 141–219. 10.1002/cphy.c100069 PMC3919066 23728973

[erag402-B71] Laureano-Marín AM, Moreno I, Romero LC, Gotor C. 2016. Negative regulation of autophagy by sulfide is independent of reactive oxygen species. Plant Physiology 171, 1378–1391. 10.1104/pp.16.00110 PMC4902596 27208225

[erag402-B72] Laureano-Marín AM, Aroca A, Pérez-Pérez ME, Yruela I, Jurado-Flores A, Moreno I, Crespo JL, Romero LC, Gotor C. 2020. Abscisic acid-triggered persulfidation of the Cys protease ATG4 mediates regulation of autophagy by sulfide. The Plant Cell 32, 3902–3920. 10.1105/tpc.20.00766 PMC7721334 33037147

[erag402-B73] Li G, Wu J, Kronzucker HJ, Li B, Shi W. 2024. Physiological and molecular mechanisms of plant-root responses to iron toxicity. Journal of Plant Physiology 297, 154257. 10.1016/j.jplph.2024.154257 38688043

[erag402-B74] Li J, Shi C, Wang X, Liu C, Ding X, Ma P, Wang X, Jia H. 2020. Hydrogen sulfide regulates the activity of antioxidant enzymes through persulfidation and improves the resistance of tomato seedling to copper oxide nanoparticles (CuO NPs)-induced oxidative stress. Plant Physiology and Biochemistry 156, 257–266. 10.1016/j.plaphy.2020.09.020 32979798

[erag402-B75] Li L, Wei S, Shen W. 2020. The role of methane in plant physiology: a review. Plant Cell Reports 39, 171–179. 10.1007/s00299-019-02478-y 31646372

[erag402-B76] Li Q, Lancaster JR. 2013. Chemical foundations of hydrogen sulfide biology. Nitric Oxide 35, 21–34. 10.1016/j.niox.2013.07.001 PMC3843984 23850631

[erag402-B77] Liu H, Wang J, Liu J, Liu T, Xue S. 2021. Hydrogen sulfide (H_2_S) signaling in plant development and stress responses. aBIOTECH 2, 32–63. 10.1007/s42994-021-00035-4 PMC7917380 34377579

[erag402-B78] Liu J, Hou Z-H, Liu G-H, Hou L-X, Liu X. 2012. Hydrogen sulfide may function downstream of nitric oxide in ethylene-induced stomatal closure in *Vicia faba* L. Journal of Integrative Agriculture 11, 1644–1653.

[erag402-B79] Liu Z, Li Y, Cao C, Liang S, Ma Y, Liu X, Pei Y. 2019. The role of H_2_S in low temperature-induced cucurbitacin C increases in cucumber. Plant Molecular Biology 99, 535–544. 10.1007/s11103-019-00834-w 30707394

[erag402-B80] Liu Z, Liu Y, Liao W. 2024. Hydrogen sulfide in the oxidative stress response of plants: crosstalk with reactive oxygen species. International Journal of Molecular Sciences 25, 1935. 10.3390/ijms25031935 PMC10856001 38339212

[erag402-B81] Lohninger L, Tomasova L, Praschberger M, Hintersteininger M, Erker T, Gmeiner BMK, Laggner H. 2015. Hydrogen sulphide induces HIF-1α and Nrf2 in THP-1 macrophages. Biochimie 112, 187–195. 10.1016/j.biochi.2015.03.009 25795259

[erag402-B82] Loreti E, Perata P. 2023. ERFVII transcription factors and their role in the adaptation to hypoxia in Arabidopsis and crops. Frontiers in Genetics 14, 1213839. 10.3389/fgene.2023.1213839 PMC10469677 37662843

[erag402-B83] Ma Y, Li F, Yi Y, et al 2023. Hydrogen sulfide improves salt tolerance through persulfidation of PMA1 in Arabidopsis. Plant Cell Reports 42, 1265–1277. 10.1007/s00299-023-03029-2 37179518

[erag402-B84] Machingura M, Salomon E, Jez JM, Ebbs SD. 2016. The β-cyanoalanine synthase pathway: beyond cyanide detoxification. Plant, Cell & Environment 39, 2329–2341. 10.1111/pce.12755 27116378

[erag402-B85] Marutani E, Morita M, Hirai S, et al 2021. Sulfide catabolism ameliorates hypoxic brain injury. Nature Communications 12, 3108. 10.1038/s41467-021-23363-x PMC8149856 34035265

[erag402-B86] Masson N, Keeley TP, Giuntoli B, White MD, Puerta ML, Perata P, Hopkinson RJ, Flashman E, Licausi F, Ratcliffe PJ. 2019. Conserved N-terminal cysteine dioxygenases transduce responses to hypoxia in animals and plants. Science 365, 65–69. 10.1126/science.aaw0112 PMC6715447 31273118

[erag402-B87] May PM, Batka D, Hefter G, Königsberger E, Rowland D. 2018. Goodbye to S^2−^ in aqueous solution. Chemical Communications 54, 1980–1983. 10.1039/c8cc00187a 29404555

[erag402-B88] Metzen E, Zhou J, Jelkmann W, Fandrey J, Brüne B. 2003. Nitric oxide impairs normoxic degradation of HIF-1α by inhibition of prolyl hydroxylases. Molecular Biology of the Cell 14, 3470–3481. 10.1091/mbc.E02-12-0791 PMC181582 12925778

[erag402-B89] Mishanina TV, Libiad M, Banerjee R. 2015. Biogenesis of reactive sulfur species for signaling by hydrogen sulfide oxidation pathways. Nature Chemical Biology 11, 457–464. 10.1038/nchembio.1834 PMC4818113 26083070

[erag402-B90] Moseler A, Wagner S, Meyer AJ. 2024. Protein persulfidation in plants: mechanisms and functions beyond a simple stress response. Biological Chemistry 405, 547–566. 10.1515/hsz-2024-0038 39303198

[erag402-B91] Mukherjee S, Corpas FJ. 2020. Crosstalk among hydrogen sulfide (H_2_S), nitric oxide (NO) and carbon monoxide (CO) in root-system development and its rhizosphere interactions: a gaseous interactome. Plant Physiology and Biochemistry 155, 800–814. 10.1016/j.plaphy.2020.08.020 32882618

[erag402-B92] Mustafa AK, Gadalla MM, Sen N, Kim S, Mu W, Gazi SK, Barrow RK, Yang G, Wang R, Snyder SH. 2009a. H_2_S signals through protein S-sulfhydration. Science Signaling 2, ra72. 10.1126/scisignal.2000464 PMC2998899 19903941

[erag402-B93] Mustafa AK, Gadalla MM, Snyder SH. 2009b. Signaling by gasotransmitters. Science Signaling 2, re2. 10.1126/scisignal.268re2 PMC2744355 19401594

[erag402-B94] Noctor G, Queval G, Mhamdi A, Chaouch S, Foyer CH. 2011. Glutathione. The Arabidopsis Book 9, e0142. 10.1199/tab.0142 PMC3267239 22303267

[erag402-B95] Olson KR . 2012. A practical look at the chemistry and biology of hydrogen sulfide. Antioxidants & Redox Signaling 17, 32–44. 10.1089/ars.2011.4401 PMC3342559 22074253

[erag402-B96] Olson KR . 2015. Hydrogen sulfide as an oxygen sensor. Antioxidants & Redox Signaling 22, 377. 10.1089/ars.2014.5930 PMC4307036 24801248

[erag402-B97] Olson KR . 2021. A case for hydrogen sulfide metabolism as an oxygen sensing mechanism. Antioxidants 10, 1650. 10.3390/antiox10111650 PMC8615108 34829521

[erag402-B98] Paul BD, Snyder SH. 2012. H_2_S signalling through protein sulfhydration and beyond. Nature Reviews. Molecular Cell Biology 13, 499–507. 10.1038/nrm3391 22781905

[erag402-B99] Paul D, Snyder SH, Kashfi K. 2021. Effects of hydrogen sulfide on mitochondrial function and cellular bioenergetics. Redox Biology 38, 101772. 10.1016/j.redox.2020.101772 PMC7606857 33137711

[erag402-B100] Peng R, Bian Z, Zhou L, Cheng W, Hai N, Yang C, Yang T, Wang X, Wang C. 2016. Hydrogen sulfide enhances nitric oxide-induced tolerance of hypoxia in maize (*Zea mays* L.). Plant Cell Reports 35, 2325–2340. 10.1007/s00299-016-2037-4 27516180

[erag402-B101] Peng Y-J, Nanduri J, Raghuraman G, Souvannakitti D, Gadalla MM, Kumar GK, Snyder SH, Prabhakar NR. 2010. H_2_S mediates O_2_ sensing in the carotid body. Proceedings of the National Academy of Sciences, USA 107, 10719–10724. 10.1073/pnas.1005866107 PMC2890835 20556885

[erag402-B102] Pereira ER, Frudd K, Awad W, Hendershot LM. 2014. Endoplasmic reticulum (ER) stress and hypoxia response pathways interact to potentiate hypoxia-inducible factor 1 (HIF-1) transcriptional activity on targets like vascular endothelial growth factor (VEGF). Journal of Biological Chemistry 289, 3352–3364. 10.1074/jbc.M113.507194 PMC3916539 24347168

[erag402-B103] Pol A, Renkema GH, Tangerman A, et al 2018. Mutations in SELENBP1, encoding a novel human methanethiol oxidase, cause extraoral halitosis. Nature Genetics 50, 120–129. 10.1038/s41588-017-0006-7 PMC5742538 29255262

[erag402-B104] Pucciariello C, Perata P. 2017. New insights into reactive oxygen species and nitric oxide signalling under low oxygen in plants. Plant, Cell & Environment 40, 473–482. 10.1111/pce.12715 26799776

[erag402-B105] Rennenberg H, Arabatzis N, Grundel I. 1987. Cysteine desulphydrase activity in higher plants: evidence for the action of L- and D-cysteine specific enzymes. Phytochemistry 26, 1583–1589.

[erag402-B106] Renziehausen T, Frings S, Schmidt-Schippers R. 2024. ‘Against all floods’: plant adaptation to flooding stress and combined abiotic stresses. The Plant Journal 117, 1836–1855. 10.1111/tpj.16614 38217848

[erag402-B107] Renziehausen T, Dirr A, Schmidt-Schippers R, Flashman E, Schippers J. 2025. Oxygen sensing and plant adaptation to flooding in a changing climate. Philosophical Transactions of the Royal Society B: Biological Sciences 380, 20240238. 10.1098/rstb.2024.0238 PMC12121377 40439298

[erag402-B108] Romero LC, Aroca MA, Laureano-Marín AM, Moreno I, García I, Gotor C. 2014. Cysteine and cysteine-related signaling pathways in *Arabidopsis thaliana*. Molecular Plant 7, 264–276. 10.1093/mp/sst168 24285094

[erag402-B109] Romero LC, Carrillo R, Montesinos-Pereira D, Luque C, Aroca A, Gotor C. 2026. Protein persulfidation: a ubiquitous modification regulating a broad spectrum of biological processes. Journal of Experimental Botany 77, 3990–4003. 10.1093/jxb/eraf464 PMC13351730 41108177

[erag402-B110] Ruetz M, Kumutima J, Lewis BE, Filipovic MR, Lehnert N, Stemmler TL, Banerjee R. 2017. A distal ligand mutes the interaction of hydrogen sulfide with human neuroglobin. Journal of Biological Chemistry 292, 6512–6528. 10.1074/jbc.M116.770370 PMC5399104 28246171

[erag402-B111] Sasidharan R, Hartman S, Liu Z, Martopawiro S, Sajeev N, Van Veen H, Yeung E, Voesenek LACJ. 2018. Signal dynamics and interactions during flooding stress. Plant Physiology 176, 1106–1117. 10.1104/pp.17.01232 PMC5813540 29097391

[erag402-B112] Scuffi D, Álvarez C, Laspina N, Gotor C, Lamattina L, García-Mata C. 2014. Hydrogen sulfide generated by L-cysteine desulfhydrase acts upstream of nitric oxide to modulate abscisic acid-dependent stomatal closure. Plant Physiology 166, 2065–2076. 10.1104/pp.114.245373 PMC4256879 25266633

[erag402-B113] Shen J, Zhan J, Zhou M, et al 2020. Persulfidation-based modification of cysteine desulfhydrase and the NADPH oxidase RBOHD controls guard cell abscisic acid signaling. The Plant Cell 32, 1000–1017. 10.1105/tpc.19.00826 PMC7145499 32024687

[erag402-B114] Smith HM, Pluth MD. 2023. Advances and opportunities in H_2_S measurement in chemical biology. JACS Au 3, 2677–2691. 10.1021/jacsau.3c00427 PMC10598833 37885594

[erag402-B115] Sun C, Yao G-F, Li L-X, Li T-T, Zhao Y-Q, Hu K-D, Zhang C, Zhang H. 2023. E3 ligase BRG3 persulfidation delays tomato ripening by reducing ubiquitination of the repressor WRKY71. Plant Physiology 192, 616–632. 10.1093/plphys/kiad070 PMC10152667 36732924

[erag402-B116] Telara Y, Akter S, Aroca A, et al 2025. Hydrogen sulfide modulates plant hypoxic responses through the persulfidation of plant cysteine oxidases. bioRxiv doi: 10.1101/2025.11.05.686772. [Preprint].

[erag402-B117] Theissen U, Hoffmeister M, Grieshaber M, Martin W. 2003. Single eubacterial origin of eukaryotic sulfide:quinone oxidoreductase, a mitochondrial enzyme conserved from the early evolution of eukaryotes during anoxic and sulfidic times. Molecular Biology and Evolution 20, 1564–1574. 10.1093/molbev/msg174 12832624

[erag402-B118] Tiranti V, Viscomi C, Hildebrandt T, et al 2009. Loss of ETHE1, a mitochondrial dioxygenase, causes fatal sulfide toxicity in ethylmalonic encephalopathy. Nature Medicine 15, 200–205. 10.1038/nm.1907 19136963

[erag402-B119] Ugalde JM, Aller I, Kudrjasova L, et al 2022. Endoplasmic reticulum oxidoreductin provides resilience against reductive stress and hypoxic conditions by mediating luminal redox dynamics. The Plant Cell 34, 4007–4027. 10.1093/plcell/koac202 PMC9516139 35818121

[erag402-B120] Vignane T, Filipovic MR. 2023. Emerging chemical biology of protein persulfidation. Antioxidants & Redox Signaling 39, 19–39. 10.1089/ars.2023.0352 PMC10433728 37288744

[erag402-B121] Vitvitsky V, Kabil O, Banerjee R. 2012. High turnover rates for hydrogen sulfide allow for rapid regulation of its tissue concentrations. Antioxidants & Redox Signaling 17, 22–31. 10.1089/ars.2011.4310 PMC3342560 22229551

[erag402-B122] Vitvitsky V, Yadav PK, Kurthen A, Banerjee R. 2015. Sulfide oxidation by a noncanonical pathway in red blood cells generates thiosulfate and polysulfides. Journal of Biological Chemistry 290, 8310–8320. 10.1074/jbc.M115.639831 PMC4375485 25688092

[erag402-B123] Vitvitsky V, Miljkovic JL, Bostelaar T, et al 2018. Cytochrome c reduction by H_2_S potentiates sulfide signaling. ACS Chemical Biology 13, 2300–2307. 10.1021/acschembio.8b00463 PMC6450078 29966080

[erag402-B124] Voesenek LACJ, Bailey-Serres J. 2015. Flood adaptive traits and processes: an overview. New Phytologist 206, 57–73. 10.1111/nph.13209 25580769

[erag402-B125] Vojtovič D, Luhová L, Petřivalský M. 2021. Something smells bad to plant pathogens: production of hydrogen sulfide in plants and its role in plant defence responses. Journal of Advanced Research 27, 199–209. 10.1016/j.jare.2020.09.005 PMC7728587 33318878

[erag402-B126] Wang C, Deng Y, Liu Z, Liao W, Corpas FJ, Li J, Kovacik J. 2021. Hydrogen sulfide in plants: crosstalk with other signal molecules in response to abiotic stresses. International Journal of Molecular Sciences 22, 12068. 10.3390/ijms222112068 PMC8585011 34769505

[erag402-B127] Wang L, Mu X, Chen X, Han Y. 2022. Hydrogen sulfide attenuates intracellular oxidative stress via repressing glycolate oxidase activities in *Arabidopsis thaliana*. BMC Plant Biology 22, 98. 10.1186/s12870-022-03490-3 PMC8897949 35247968

[erag402-B128] Wang R . 2002. Two’s company, three’s a crowd: can H_2_S be the third endogenous gaseous transmitter? The FASEB Journal 16, 1792–1798. 10.1096/fj.02-0211hyp 12409322

[erag402-B129] Wang R . 2012. Physiological implications of hydrogen sulfide: a whiff exploration that blossomed. Physiological Reviews 92, 791–896. 10.1152/physrev.00017.2011 22535897

[erag402-B130] Wang R . 2014. Gasotransmitters: growing pains and joys. Trends in Biochemical Sciences 39, 227–232. 10.1016/j.tibs.2014.03.003 24767680

[erag402-B131] Wang XX, Shi C, Hu Y, et al 2023. Persulfidation maintains cytosolic G6PDs activity through changing tetrameric structure and competing cysteine sulfur oxidation under salt stress in Arabidopsis and tomato. New Phytologist 240, 626–643. 10.1111/nph.19188 37574819

[erag402-B132] Watanabe M, Kusano M, Oikawa A, Fukushima A, Noji M, Saito K. 2008. Physiological roles of the beta-substituted alanine synthase gene family in Arabidopsis. Plant Physiology 146, 310–320. 10.1104/pp.107.106831 PMC2230570 18024555

[erag402-B133] Wedmann R, Onderka C, Wei S, et al 2016. Improved tag-switch method reveals that thioredoxin acts as depersulfidase and controls the intracellular levels of protein persulfidation. Chemical Science 7, 3414–3426. 10.1039/c5sc04818d PMC4845716 27170841

[erag402-B134] Weits DA, Giuntoli B, Kosmacz M, Parlanti S, Hubberten H, Riegler H, Hoefgen R, Perata P, Van Dongen JT, Licausi F. 2014. Plant cysteine oxidases control the oxygen-dependent branch of the N-end-rule pathway. Nature Communications 5, 3425. 10.1038/ncomms4425 PMC3959200 24599061

[erag402-B135] Wilson LG, Bressan RA, Filner P. 1978. Light-dependent emission of hydrogen sulfide from plants. Plant Physiology 61, 184–189. 10.1104/pp.61.2.184 PMC1091829 16660257

[erag402-B136] Wu B, Teng H, Yang G, Wu L, Wang R. 2012. Hydrogen sulfide inhibits the translational expression of hypoxia-inducible factor-1α. British Journal of Pharmacology 167, 1492–1505. 10.1111/j.1476-5381.2012.02113.x PMC3514762 22831549

[erag402-B137] Wu B, Teng H, Zhang L, Li H, Li J, Wang L, Li H. 2015. Interaction of hydrogen sulfide with oxygen sensing under hypoxia. Oxidative Medicine and Cellular Longevity 2015, 758678. 10.1155/2015/758678 PMC4442289 26078818

[erag402-B138] Xiao Y, Wu X, Sun M, Peng F. 2020. Hydrogen sulfide alleviates waterlogging-induced damage in peach seedlings via enhancing antioxidative system and inhibiting ethylene synthesis. Frontiers in Plant Science 11, 696. 10.3389/fpls.2020.00696 PMC7274156 32547587

[erag402-B139] Xu F, Peng Y, He Z-Q, Yu L-L. 2023. The role of cyanoalanine synthase and alternative oxidase in promoting salt stress tolerance in *Arabidopsis thaliana*. BMC Plant Biology 23, 163. 10.1186/s12870-023-04167-1 PMC10041793 36973660

[erag402-B140] Xuan L, Li J, Wang X, Wang C. 2020. Crosstalk between hydrogen sulfide and other signal molecules regulates plant growth and development. International Journal of Molecular Sciences 21, 4593. 10.3390/ijms21134593 PMC7370202 32605208

[erag402-B141] Xuan L, Wu H, Li J, Yuan G, Huang Y, Lian C, Wang X, Yang T, Wang C. 2022. Hydrogen sulfide reduces cell death through regulating autophagy during submergence in Arabidopsis. Plant Cell Reports 41, 1531–1548. 10.1007/s00299-022-02872-z 35507055

[erag402-B142] Yang M, Wei J, Xu Y, et al 2025. Autophagy regulates plant tolerance to submergence by modulating photosynthesis. Plant, Cell & Environment 48, 2267–2284. 10.1111/pce.15290 39575938

[erag402-B143] Yang T, Yuan G, Zhang Q, Xuan L, Li J, Zhou L, Shi H, Wang X, Wang C. 2021. Transcriptome and metabolome analyses reveal the pivotal role of hydrogen sulfide in promoting submergence tolerance in Arabidopsis. Environmental and Experimental Botany 183, 104365.

[erag402-B144] Yao Y, Yang Y, Li C, Huang D, Zhang J, Wang C, Li W, Wang N, Deng Y, Liao W. 2019. Research progress on the functions of gasotransmitters in plant responses to abiotic stresses. Plants (Basel, Switzerland) 8, 605. 10.3390/plants8120605 PMC6963697 31847297

[erag402-B145] Yuan J, Zhao X, Zhong Y, Wu X, Wei S, Wei L, Zhu Y, Liu Y. 2025. The roles of hydrogen sulfide in maintaining postharvest fruit quality: crosstalk with phytohormones. Food Research International 212, 116452. 10.1016/j.foodres.2025.116452 40382044

[erag402-B146] Yuan L-B, Dai Y-S, Xie L-J, Yu L-J, Zhou Y, Lai Y-X, Yang Y-C, Xu L, Chen Q-F, Xiao S. 2017. Jasmonate regulates plant responses to postsubmergence reoxygenation through transcriptional activation of antioxidant synthesis. Plant Physiology 173, 1864–1880. 10.1104/pp.16.01803 PMC5338657 28082717

[erag402-B147] Zaffagnini M, Bedhomme M, Groni H, Marchand CH, Puppo C, Gontero B, Cassier-Chauvat C, Decottignies P, Lemaire SD. 2012. Glutathionylation in the photosynthetic model organism *Chlamydomonas reinhardtii*: a proteomic survey. Molecular & Cellular Proteomics 11, M111.014142. 10.1074/mcp.M111.014142 PMC3277769 22122882

[erag402-B148] Zaffagnini M, Fermani S, Costa A, Lemaire SD, Trost P. 2013. Plant cytoplasmic GAPDH: redox post-translational modifications and moonlighting properties. Frontiers in Plant Science 4, 450. 10.3389/fpls.2013.00450 PMC3824636 24282406

[erag402-B149] Zhang J, Zhang L, Zhang W, et al 2025. H_2_S activates succinate dehydrogenase through persulfidation to induce stomatal closure in Arabidopsis. Plant and Soil 508, 939–954.

[erag402-B150] Zhong Y-H, Guo Z-J, Wei M-Y, et al 2023. Hydrogen sulfide upregulates the alternative respiratory pathway in mangrove plant *Avicennia marina* to attenuate waterlogging-induced oxidative stress and mitochondrial damage in a calcium-dependent manner. Plant, Cell & Environment 46, 1521–1539. 10.1111/pce.14546 36658747

[erag402-B151] Zhou H, Zhang J, Shen J, Zhou M, Yuan X, Xie Y. 2020. Redox-based protein persulfidation in guard cell ABA signaling. Plant Signaling & Behavior 15, 1741987. 10.1080/15592324.2020.1741987 PMC7238880 32178559

[erag402-B152] Zhou L, Gao X, Weits DA, Zeng P, Wang X, Cai J. 2021. H_2_S regulates low oxygen signaling via integration with the unfolded protein response in *Arabidopsis thaliana*. Plant and Soil 467, 531–547.

[erag402-B153] Zhou M, Xie Y, Van Breusege F, Huang J. 2025. Hydrogen sulfide and protein persulfidation in plant stress signaling. Journal of Experimental Botany 76, 3738–3757. 10.1093/jxb/eraf100 40059712

[erag402-B154] Zivanovic J, Kouroussis E, Kohl JB, et al 2019. Selective persulfide detection reveals evolutionarily conserved antiaging effects of S-sulfhydration. Cell Metabolism 30, 1152–1170.e13. 10.1016/j.cmet.2019.10.007 PMC7185476 31735592

